# Chromosome-level genome assembly of Asian yellow pond turtle (*Mauremys mutica*) with temperature-dependent sex determination system

**DOI:** 10.1038/s41598-022-12054-2

**Published:** 2022-05-12

**Authors:** Xiaoli Liu, Yakun Wang, Ju Yuan, Fang Liu, Xiaoyou Hong, Lingyun Yu, Chen Chen, Wei Li, Wei Ni, Haiyang Liu, Jian Zhao, Chengqing Wei, Haigang Chen, Yihui Liu, Xinping Zhu

**Affiliations:** 1 Key Laboratory of Tropical and Subtropical Fishery Resource Application and Cultivation of Ministry of Agriculture and Rural Affairs, Pearl River Fisheries Research Institute, Chinese Academy of Fishery Sciences, Guangzhou, 51038 China; 2 College of Life Science and Fisheries, Shanghai Ocean University, Shanghai, 201306 China

**Keywords:** Genetics, Molecular biology

## Abstract

Knowledge of sex determination has important implications in physiology, ecology and genetics, but the evolutionary mechanisms of sex determination systems in turtles have not been fully elucidated, due to a lack of reference genomes. Here, we generate a high-quality genome assembly of Asian yellow pond turtle (*Mauremys mutica*) using continuous long-read (PacBio platform), Illumina, and high-throughput chromatin conformation capture (Hi-C) technologies. The *M*. *mutica* haplotype has a genome size of 2.23 Gb with a contig N50 of 8.53 Mb and scaffold N50 of 141.98 Mb. 99.98% sequences of the total assembly are anchored to 26 pseudochromosomes. Comparative genomics analysis indicated that the lizard-snake-tuatara clade diverged from the bird-crocodilian-turtle clade at approximately 267.0–312.3 Mya. Intriguingly, positive selected genes are mostly enriched in the calcium signaling pathway and neuroactive ligand-receptor interaction, which are involved in the process of temperature-dependent sex determination. These findings provide important evolutionary insights into temperature-dependent sex determination system.

## Introduction

Sex has been revealed to have significant implications for physiology and evolutionary biology by driving beneficial mutations, altering genetic complexity and increasing environmental adaptation^1,2^. Sex determination is the developmental decision of an undifferentiated primordial gonad into a testis or ovary, while sex differentiation is the biological process that differentiates into male or female after sex determination^3^. Sex systems of most gonochoristic vertebrates fall into two categories: genotypic sex determination (GSD) and environmental sex determination (ESD)^4,5^. In GSD animals, such as mammals^6^, birds^7^, amphibians^8^, some reptiles^9^ and most fishes^10^, the initial sex is highly determined by genotypic elements carried by sperm and ovum at the time of fertilization. For ESD species, there is no genetic difference between the sexes, and sex development is triggered by external stimuli, such as temperature^11^, humidity^12^, photoperiod^13^ and social factors^13^.

Temperature-dependent sex determination (TSD) is the most typical class of ESD, in which the percentage of male or female offspring is determined by the ambient temperature during early embryo or larva development in some reptiles and fishes^14–17^. The investigation on TSD model was firstly reported in a lizard *Agama agama* by the French zoologist Madeleine Charnier^18^. However, her research has been questioned for a long time as most biologists believe that TSD is merely a defect in the sexual development of reptiles^19^ or a substitute mode of GSD^20^. It was not until 1979 that the research on the TSD model really began, thanks to Bull and Vogt's demonstration of the effect of temperature on the sex ratio of five turtles using laboratory and field data^21^. Recently, great progress in elucidating the TSD mechanism has been made in red-eared slider turtle (*Trachemys scripta*)^11,22,23^. Moreover, among the published five turtle species including *T. scripta*, *Chrysemys picta*, *Chelonia mydas*, *Platysternon megacephalum*, and *Pelodiscus sinensis*^24–26^, only the genome of *T*. *scripta* has been assembled at the chromosome level.

The Asian yellow pond turtle, *Mauremys mutica*, an important freshwater turtle species, belongs to the family Geoemydidae and is widely distributed in eastern and southern China and northern Vietnam and Japan^27–29^. Obvious sexually dimorphic modifications at sexual maturity have been reported in previous studies^30^. Briefly, these include (a) males growing faster than females; (b) the male's carapace is concave to prevent it from sliding off the female's shell during mating; (c) male tails are longer and stronger than that of females, and the male cloacae are farther from the base of the tails than in females. In addition, the sex of *M. mutica* is determined by incubation temperature with no heteromorphic sex chromosomes or sex-specific genetic marker have been detected^31^. The TSD patterns are different between *M. mutica* and *T*. *scripta* that low/high temperatures lead to all-male/-female embryos in *T*. *scripta* while low/high temperatures result in high proportion-male/-female embryos in *M. mutica*, indicating diverse TSD mechanisms between these turtle species.

**Figure 1:**
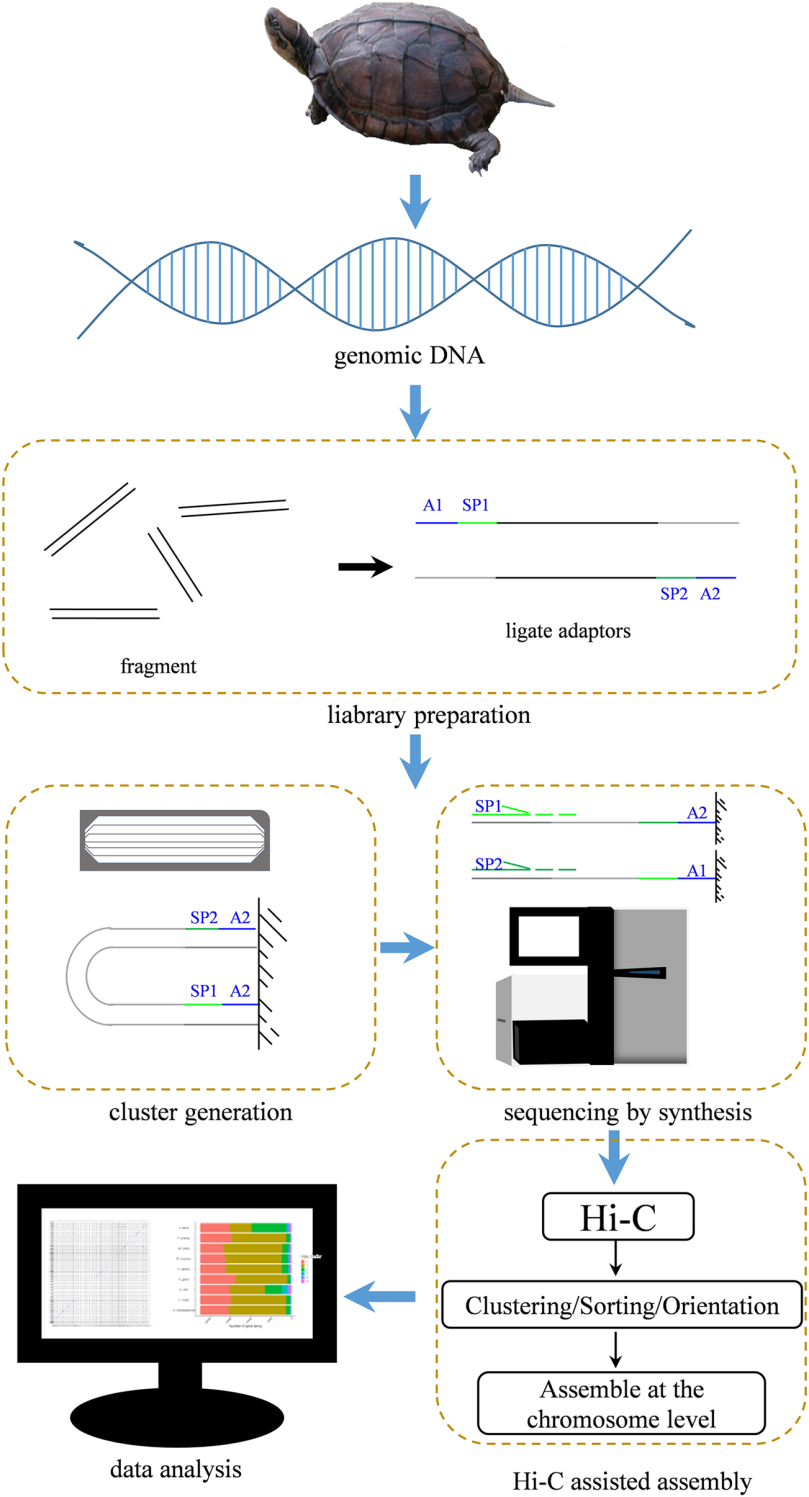
Workflow of the Asian yellow pond turtle sampling, DNA extraction, genome sequencing, assembly and data analysis. Green lines (SP1 and SP2) represent adapters.

Here, we present a high-quality genome assembly of *M. mutica* at the chromosome level, via a combination of continuous long-read (PacBio platform), Illumina, and high-throughput chromatin conformation capture (Hi-C) technologies (Fig. 1). The high-quality reference genome constructed in this study will be of benefit for elucidating the genetic mechanism underlying sex determination and gonadal development in TSD *M. mutica*.

## Material and methods

### Sample preparation and genome sequencing

A healthy female *M. mutica* (estimated age 4 years) was obtained from Guangzhou aquatic thoroughbred base of the Pearl River Fisheries Research Institute. Liver and muscle tissues were flash frozen in liquid nitrogen, and genomic DNA was extracted using a DNeasy Blood and Tissue Kit (Qiagen, Valencia, CA, USA) according to the manufacturer’s instructions. DNA quantity and quality were measured using Qubit 3.0 and 1% agarose gel electrophoresis, respectively. High-quality DNA was used for continuous long-read library construction and sequencing in PacBio platform. Then pair-ended libraries and mate-paired libraries were prepared using the standard Illumina protocol. Library sequencing was performed using the Illumina HiSeq4000 platform (Illumina, San Diego, CA, USA) to further evaluate the PacBio assembly quality (Fig. 1). After discarding low-quality reads, adapter sequences, and contaminant reads, including mitochondrial DNA, plant, bacterial, and viral sequences, clean reads were used for subsequent genome survey, correction, and evaluation. We declare that all animal experiments in this research were performed according to the guidelines established by the Pearl River Fisheries Research Institute, Chinese Academy of Fishery Sciences. Turtles used were treated humanely and ethically, and the experiments were approved by Laboratory Animal Ethics Committee Pearl River Fisheries Research Institute, Chinese Academy of Fishery Sciences.

### Genome size estimation

A *k*-mer depth frequency distribution analysis was performed to estimate the genome size, heterozygosity and repetitive sequences^32^. Under the premise of uniform distribution of sequencing reads, genome size (G) was evaluated based on the following formula: G = *k*-mer number/mean *k*-mer depth.

### Hi-C library construction and sequencing

To further obtain a chromosomal-level assembly of the genome, a Hi-C library was created for sequencing of adult female liver tissue. The liver tissue sample was fixed in paraformaldehyde, and DNA molecules were enzymatically digested with *Mbo*I, generating sticky ends. After repairing and labeling the 5′ overhang with biotinylated residue, the DNA fragments were ligated to each other to form chimeric circles using DNA ligase. Biotinylated circles, which are chimeras of physically associated DNA molecules from the original cross-linking, were enriched, sheared, and sequenced on the Illumina HiSeq X10 platform (San Diego, CA, United States) in 150 PE mode.

### Chromosome assembly using Hi-C data

Illumina reads (119×) and PacBio long reads (10×) should corrected to obtain high-accuracy data. Clean data was corrected using Canu 1.5^33^. After assembly of the corrected subreads using SMARTdenovo software, the draft genome was polished with 50× Illumina short reads using Pilon v1.22^34^. Subsequently, Hi-C data were performed to assembled contigs to chromosome-level scaffolds.

### Karyotype analysis

The turtle was intraperitoneally injected with 10 μg/g body weight of phytohemagglutinin (PHA), and 24 h later, injected colchicine with 5ug/g-6ug/g body weight. The spleen was taken after 3.5 h. Then, the tissues were incubated with hypotonic solution (0.0375 M KCl) for 15–20 min, and fixed twice in methanol acetic acid (3:1) for 20 min at 4 °C. The fixed tissue suspension was then dropped onto clean glass slides, air-dried, and stained with 10% Giemsa solution (10 mM potassium phosphate, pH 6.8). The chromosomes were cut out and arranged according to the following standards: Group A consists of macrochromosomes with median (M) or sub-median centromeres (SM); Group B consists of macrochromosomes with terminal centromere (T) or subterminal centromeres (ST); the Group C can be considered microchromosomes (m).

### Assessment of the genome assemblies

To evaluate the quality and completeness of the genome assembly, we first aligned the Illumina reads onto the assembly using BWA v0.7.10-r789^35^ to assess the alignment rate. Moreover, CEGMA v2.5^36^ was performed to identify conserved core eukaryotic genes (CEGs) with the parameter set as identity > 70%. Finally, BUSCO v2^37^ was used to further detect single-copy orthologs to evaluate the completeness, degree of fragmentation and missing genes of the genome assembly.

### Gene prediction and functional annotation

We constructed the de novo repeat library using LTR FINDER v1.07^38^, RepeatScout v1.0.5^39^ and PILER-DF v2.4^40^. PASTEClassifier v1.0^41^ was used to classify different types of repetitive sequences and then merged with the Repbase v.22.11^42^ library to produce the ultimate repeat library. Finally, RepeatMasker v4.0.6^43^ was applied to identify and mask the repeated sequences.

Three approaches, including ab initio prediction, homology-based search and RNA-sequencing (RNAseq)-based prediction, were integrated to annotate protein-coding genes in the *M*. *mutica* genome assembly. For ab initio prediction, five tools, Augustus v3.1^44^, GlimmerHMM v1.2^45^, GeneID v1.4^46^, SNAP v2006-07–28^47^ and Genscan v3.1^48^, were used with default settings. For homology-based searches, protein sequences of four closely related reptiles (*C*. *mydas*, *C*. *picta*, *T*. *scripta* and *P*. *sinensis*) were downloaded from the NCBI database and aligned to the assembled genome with GeMoMa v1.3.1 to determine accurately spliced alignments^49^. For RNAseq-based prediction, transcriptome data from mixed tissues, including heart, liver, spleen, kidney, brain, muscle, eye, testis and ovary, were assembled using Trinity v2.1.1^50^, followed by gene predictions with Program to Assemble Spliced Alignments (PASA) v2.0.2^51^. Finally, EVidencemodeler (EVM) v1.1.1^52^ and PASA v2.0.2 were performed to merge the prediction results obtained from these strategies.

Functional annotations of the predicted genes were performed by homology alignment to public gene databases, including Kyoto Encyclopedia of Genes and Genomes (KEGG)^53^, KOG (Clusters of orthologous groups for eukaryotic complete genomes)^54^, TrEMBL^55^, NCBI nonredundant protein sequences (NR) using BLAST v2.2.31 with an e-value threshold of 1 e−5^56^, and the GO (Gene Ontology) database using Blast2GO v2.5^57^.

Moreover, noncoding RNAs were identified by alignment to the Rfam v12.1^58^ and miRBase v21.0 databases^59^. Transfer RNAs (tRNAs) were predicted using tRNAscan-SE v1.3.1^60^, and putative ribosomal RNAs (rRNAs) and microRNAs (miRNAs) were predicted using Infernal v1.1^61^.

### Evolutionary and comparative genomic analyses

To investigate the phylogenetic relationships between *M*. *mutica* and other species, we first used Orthofinder v2.3.7^62^ to identify orthologous gene families by comparing protein data of eight other genomes from previously reported reptiles, including *C*. *picta* (GCA_000241765.2), *C*. *mydas* (GCA_015237465.1), *T*. *scripta* (GCA_013100865.1), *Platysternon megacephalum* (GCA_003942145.1), *P*. *sinensis* (GCA_000230535.1), *Anolis carolinensis* (GCA_000090745.2), *Alligator mississippiensis* (GCA_000281125.4), *Deinagkistrodon acutus*, one bird *Gallus gallus* (GCA_000002315.5) and two mammals *Mus musculus* (GCA_000001635.9) and *Homo sapiens* (GCA_000001405.28). The software MUSCLE v3.8.31^63^ was applied with default parameters to further extract single-copy orthologous genes shared among these 12 species. Shared gene families were visualized using the upsetr^64^ package as implemented in R. Subsequently, the phylogenetic tree was reconstructed based on single-copy orthologous genes using IQ-TREE v1.6.11^65^. Briefly, each single-copy gene family was aligned using MAFFT v7.205^66^, and then the alignment of proteins was converted into codon sequences using PAL2NAL v14^67^. After removing regions with poor sequence alignment or large differences by Gblocks v0.91b^68^, we concatenated well-aligned gene families into a supersequence. Then, a maximum likelihood (ML) phylogenetic tree with the GTR + F + I + G4 best-fit model and 1000 bootstrap replicates were constructed.

Moreover, divergence time was estimated using the MCMCTree package of the PAML v4.9 program^69^ under the relaxed clock model. Fossil records obtained from the TimeTree database (http://www.timetree.org/) including divergence times between *C. picta* and *T. scripta* (28.14–29.63 million years ago [Mya]), *C. picta* and *P. megacephalum* (67–79.7 million years ago [Mya]), *C. picta* and *H. sapiens* (294–323 Mya), *A. carolinensis* and *D. acutus* (156–174 Mya) were used as the calibration times. The correlated molecular clock and JC69 model were used to estimate divergence time. Parameters of Iterations of Markov Chain were as follows: burn-in = 5,000,000, sample number = 5,000,000, sample frequency = 30. The final evolutionary tree with divergence time was visualized using MCMCTreeR v1.1^70^.

### Positive selection analysis

To identify possible positively selected genes (PSGs) in the Asian yellow pond turtle genome, we first extracted the single-copy orthologous genes shared among the Asian yellow pond turtle and five turtles (*T*. *scripta*, *C. mydas*, *C. picta*, *P. megacephalum* and *P. sinensis*), and then the protein sequences of each gene family were aligned using MAFFT^66^. Subsequently, the ratio (ω) of synonymous (*K*s) and nonsynonymous (*K*a) substitutions was estimated using a branch-site model of CODEML in PAML v4.4c^69^. The likelihood of the positive selection model M2a was then compared to the null model M1a using the likelihood ratio test (LRT), and the corresponding *p*-values were calculated. Sites with ω > 1 were then calculated for posterior probability using Bayes Empirical Bayes (BEB) method, and genes with LRT* p* < 0.01 and at least one codon with a posterior probability > 0.95 were defined as PSGs. Moreover, KEGG annotations of PSGs were conducted based on functional enrichment analysis in KOBAS 3.0 (*p* < 0.05 by Fisher's exact test).

Quantitative reverse-transcription PCR (qRT-PCR) was performed to further verify the expression level of the positively selected genes. Ovaries and testes were collected from adult *M. mutica*. Total RNAs were extracted using SV Total RNA Isolation System (Promega) and then reverse-transcribed using a SuperScript™ III First-Strand Synthesis System (Invitrogen) after DNase treatment. Specific primers used were shown in Table S1. Reactions were run with the following program: 95℃ for 2 min, followed by 40 cycles of 95℃ for 15 s, 57℃ for 30 s, and 72℃ for 30 s. The *β-actin* was amplified as an internal control and the relative expression levels of positively selected genes were calculated using the 2^−ΔCt^ method. SPSS 20.0 was used to perform statistical analyses and variance at a significance level of 0.05.

### Ethics approval and consent to participate

Turtles used were treated humanely and ethically, and the experiments were approved by the Pearl River Fisheries Research Institute, Chinese Academy of Fishery Sciences. We declare that all methods were performed in accordance with ARRIVE guidelines.

## Results

### Genome sequencing and assembly

The *k*-mer (*k* = 21 analyzed here) depth frequency distribution analysis was performed to evaluate the genome size, repeat proportion and rate of heterozygosity (Fig. S1 and Table S2). The average *k*-mer depth was 53, sequences with *k*-mer depths greater than 106 were repeated sequences, and sequences with depths of approximately 26 were heterozygous. Thus, based on 21-mer frequencies, the estimated heterozygosity and repeat sequence content of the *M*. *mutica* genome were approximately 0.6% and 52.10%, respectively (Table S2). Moreover, a total of 148,757,912,763 *k*-mers were obtained from sequencing data. After removing data with abnormal depth, a total of 142,693,993,736 *k*-mers were used to further estimate the genome size; thus, we first estimated that the genome size of *M*. *mutica* was 2.69 Gb (Table S2).

**Figure 2:**
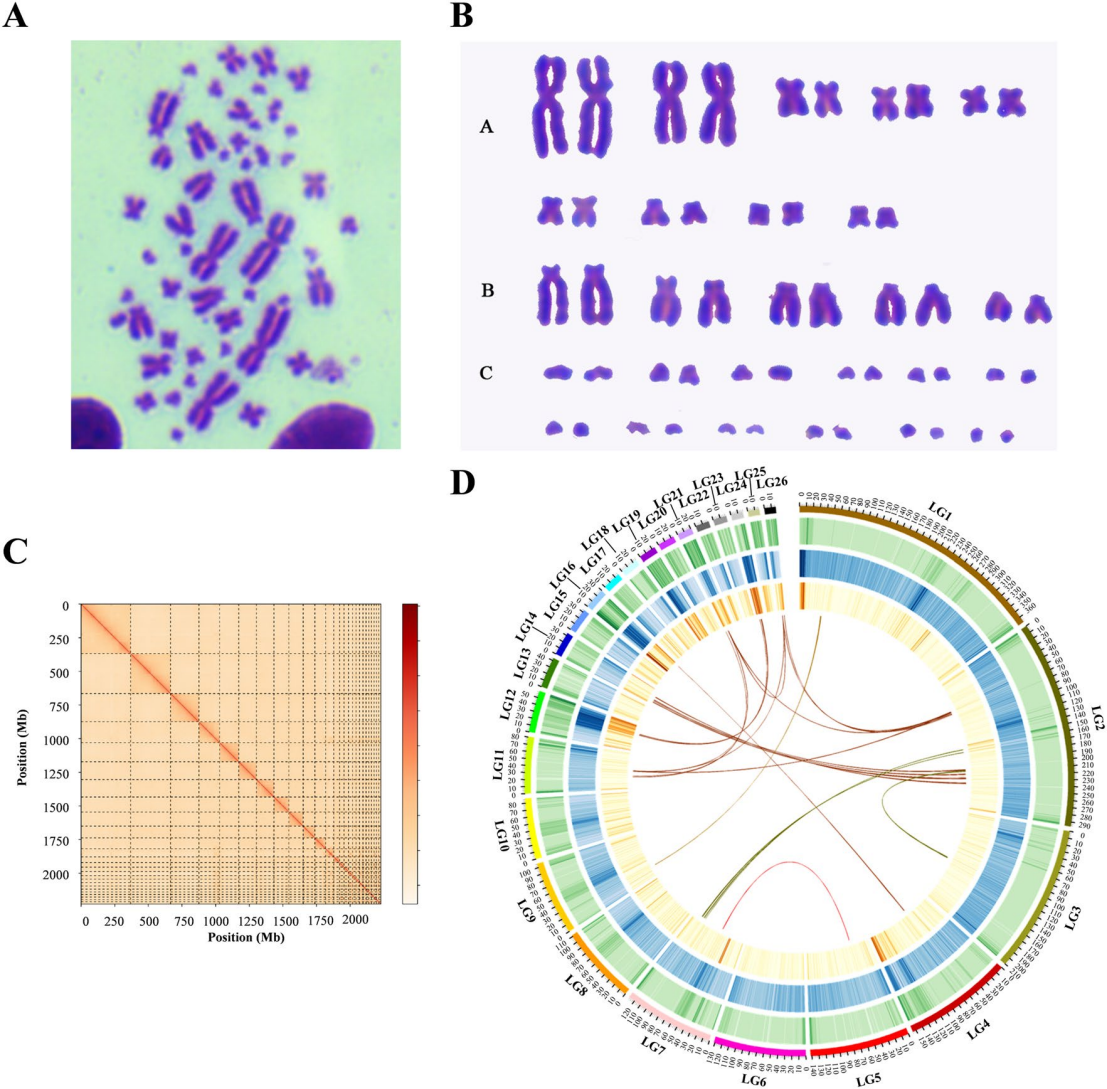
Chromosome-scale genome assembly and genomic features of the Asian yellow pond turtle. (**A**) and (**B**) Karyotype of the Asian yellow pond turtle from spleen (2n = 52). Group A is metacentric chromosome and submetacentric chromosome, group B is subtelocentric chromosome and telocentric chromosome, and group C is microchromosome. (**C**) Chromosomal interaction maps. The color from light to dark indicates an increase in the interaction intensity. The abscissa and ordinate indicate their N*bin position in the genome. The 26 squares represent the constructed 26 chromosomes of Asian yellow pond turtles. (**D**) Circos atlas of the chromosomal genome of the Asian yellow pond turtle. From outer to inner circles: (I) Asian yellow pond turtle chromosomes; (II) Distribution of GC content across the genome; (III) Repetitive sequence coverage; (IV) Gene density across the genome; and (V) Chromosome collinearity, with each line joining internal syntenic blocks.

A total of 280.42 Gb of high-quality clean data were generated from the PacBio sequencing platform (approximately 118.75×) with a read N50 of 26,874 bp and an average read length of 17,925 bp (Table S3). After Canu 1.5 correction, the data were assembled using SMARTdenovo followed by Pilon polishing, which produced 2.36 Gb total length of the genome assembly with 1530 contigs and contig N50 of 8.61 Mb (Table S4). Subsequently, LACHESIS (http://shendurelab.github.io/LACHESIS/) was used to assemble contigs at the chromosome level based on Hi-C sequencing data. The assembled genome finally resulted in 2.23 Gb, which encompassed 1106 contigs with a contig N50 of 8.53 Mb, scaffold N50 of 141.98 Mb, and anchoring rate of 94.38% (Table S4 and Table 1). This scaffold N50 is the largest compared to other sequenced turtle species (Table 1). In addition, approximately 2,211,083,089 out of 2,330,098,296 (94.89%) of length greater than 100 kb were anchored (Table S4). Chromosome integrity examination revealed that *M. mutica* were diploids with normal karyotype and chromosome number (2n = 52) (Fig. 2A,B). It was consistent with the Hi-C analysis of the genome that contained 26 pseudochromosomes (Fig. 2C,D and Table S5). Notably, 12 microchromosome-pairs were identified in *M. mutica* genome which was in accord with the prevalence of microchromosome in birds and other reptiles^71,72^. The genome-wide Hi-C heatmap of chromosome crosstalk was in accordance with the rule of interaction where the signal strength around the diagonal was obviously stronger than that of other positions, indicating the high quality and completeness of this genome assembly.

**Table 1:** Basic statistics of sequenced turtle genomes.

	*M*. *mutica*	*T*. *scripta*	*C*. *picta*	*C*. *mydas*	*P*. *megacephalum*	*P*. *sinensis*
Genome size	2.23 Gb	2.269 Gb	2.59 Gb	2.24 Gb	2.32 Gb	2.21 Gb
Sequencing depth	118.75	53.5	18	82.3	208.9	105.6
N50 scaffold	141.98 Mb	129.68 Mb	5.2 Mb	3.78 Mb	7.22 Mb	3.33 Mb

Furthermore, Illumina reads were aligned to the reference genome to further evaluate the assembly quality. Approximately 99.69% of the clean reads were mapped to the contigs, and 97.05% of the clean reads were mapped in proper pairs (Table S6). Subsequently, CEGMA v2.5 was performed to assess the completeness of the conserved core eukaryotic genes (CEGs). In total, 449 CEGs, accounting for 98.03% of all 458 CEGs, and 231 CEGs, accounting for 93.15% of 248 highly conserved CEG datasets, were identified (Table S7). Finally, BUSCO v2 was used to examine genome integrity, the degree of fragmentation, and possible loss rates. The results showed that 2,494 (96.44%) and 61 (2.36%) of the 2586 expected conserved core genes in the vertebrate database were identified as complete BUSCOs and fragmented BUSCOs, respectively, suggesting high completeness of the assembled genome and validity for subsequent analysis (Table S8).

### Genome annotation

The overall genome of *M. mutica* has a GC content of 45.11%, which is higher than that of the *T. scripta* (44.21%), *P. sinensis* (44.4%), *C. mydas* (43.5%), and *C. picta* (43%) assembled genomes (Table S4)^24,25^. Approximately 1,448,589,111 bp of repetitive sequences accounting for 61.33% of the genome assembly were identified based on the combined de novo prediction and homology search against the Repbase database (Table S9). RNA transposons (Class I) occupied approximately 45.52% of the genome content, which was higher than that of DNA transposons (Class II) (Table S9). Long interspersed nuclear elements (LINEs) were the most abundant repetitive elements, followed by terminal inverted repeats (TIRs) and penelope-like elements (PLEs) (Table S9). Moreover, several unknown repetitive sequences were also found, which constituted 2.31% of the genome assembly (Table S9).

**Figure 3:**
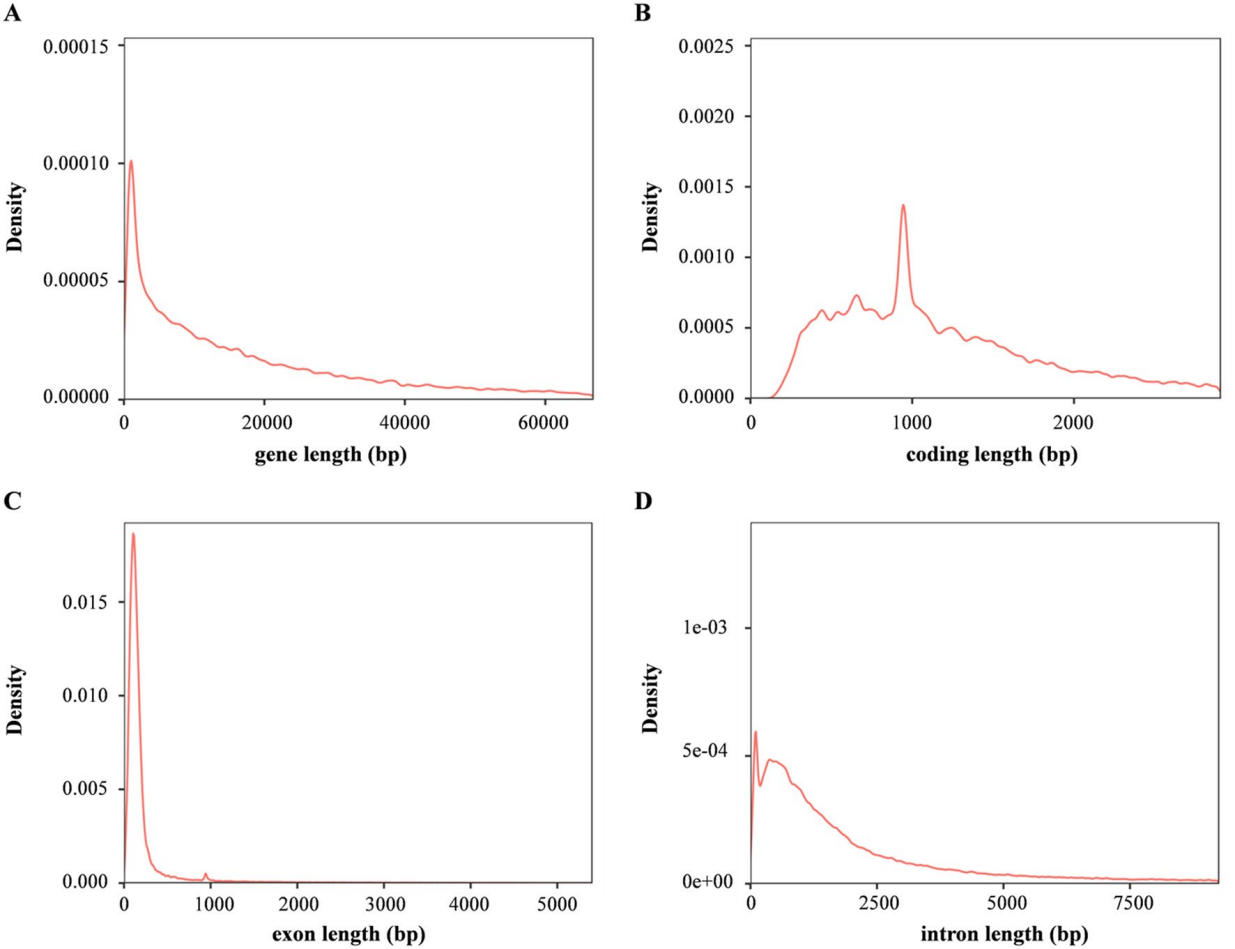
Length distribution of genes (**A**), coding sequences (**B**), exons (**C**) and introns (**D**) in *M. mutica*.

A total of 24,751 protein-coding genes were obtained in *M. mutica*, higher than those detected in soft-shell turtles (19,380) and green sea turtles (18,046)^25^ (Table S10). Across these genes, 18,126 orthogroups and 176 species-specific orthogroups were identified (Table S11). The average gene length, average coding sequence length, average exon and intron length were 26,645.17, 1521.64, 2491.72 and 24,153.44, respectively (Fig. 3 and Table S12). Among these predicted genes in *M. mutica*, 24,066 (~ 97.23%) could be functionally annotated in at least one of the databases, including GO, KEGG, KOG, TrEMBL and NR (Table S13 and Figure S2). Various nonprotein coding genes were also identified, including 219 rRNAs, 8499 tRNAs and 262 microRNA genes (Table S14).

### Genome evolution

**Figure 4:**
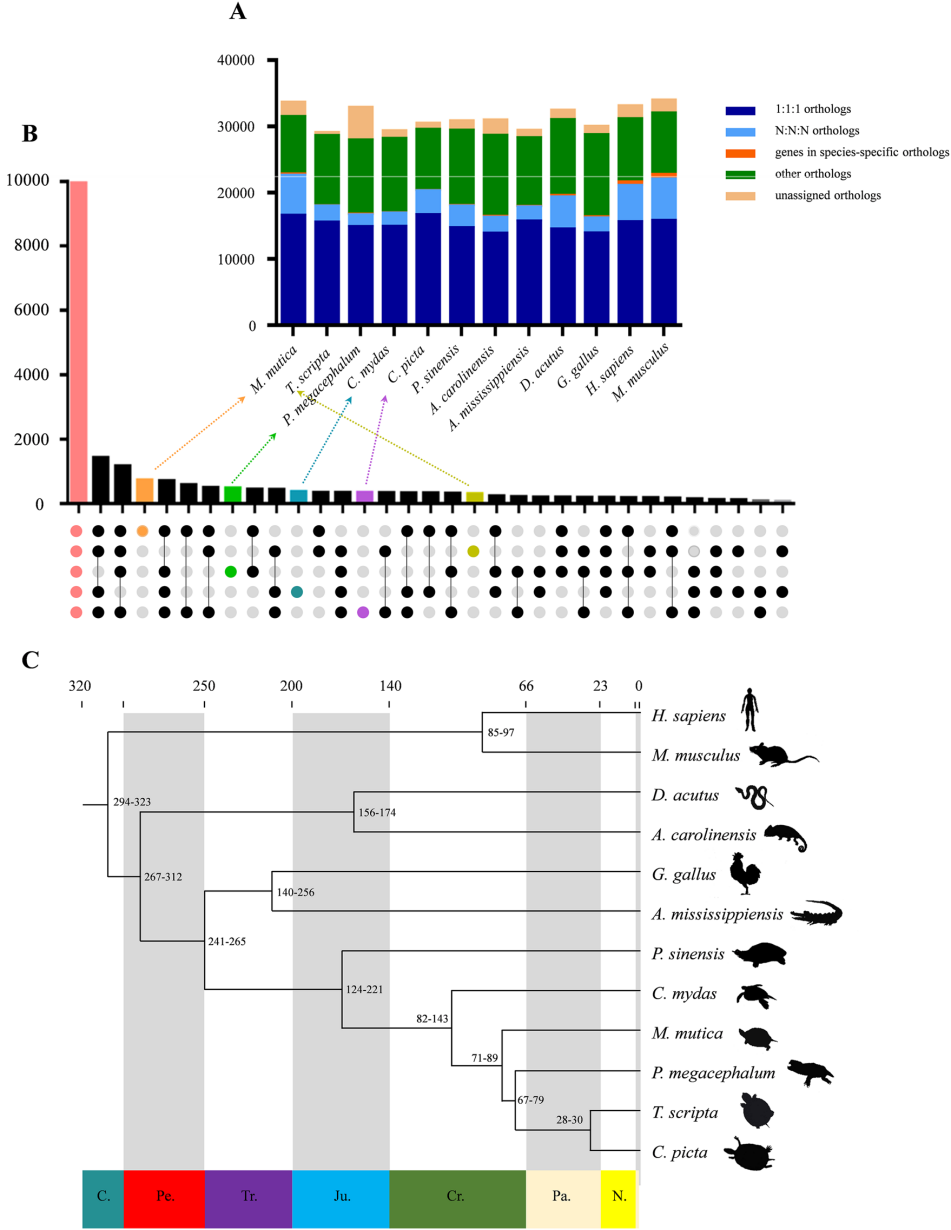
Gene family comparisons and phylogenetic analysis. (**A**) Orthologous gene families between the Asian yellow pond turtle and the other 11 vertebrates. 1:1:1 orthologs represent one copy genes in each species. N:N:N orthologous represent genes with multiple copies. (**B**) The UpSet plot of gene families in 5 turtle genomes shows shared (red), *M. mutica*-specific (orange), *P*. *megacephalum*-specific (green), *C*. *mydas*-specific (blue), *C*. *picta*-specific (purple), and *T. scripta*-specific (yellow) gene families. (**C**) Phylogenomic tree with the estimated divergence time among 12 vertebrates. The numbers on the branch represent the estimated divergence time with 95% confidence intervals. The number on the bottom of the tree is the geological time, and the number at the top of the tree is the absolute age, in millions of years, defined by the shadow of each geological period. C., Cambrian; Pe., Permian; Tr., Triassic; Ju., Jurassic; Cr., Cretaceous; Pa., Paleogene; N., Neogene.

To investigate the phylogenetic relationship of *M*. *mutica* with other groups, we compared the *M*. *mutica* genome with five other turtle species (*C*. *picta*, *C*. *mydas*, *T*. *scripta*, *P*. *megacephalum* and *P*. *sinensis*) and six other vertebrate species (*A*. *carolinensis*, *D*. *acutus*, *G*. *gallus*, *M*. *musculus*, *A*. *mississippiensis* and *H*. *sapiens*). A total of 16,484 one-to-one orthologous genes were detected in *M*. *mutica,* which was similar to *C. picta* and higher than the remaining 10 organisms (Fig. 4A). Moreover, a total of 10,179 gene families were shared by five TSD turtles (Fig. 4B). Among these, 735 M. *mutica* specific, 312 T. *scripta* specific, 485 *P*. *megacephalum* specific, 381 *C*. *mydas* specific and 347 *C*. *picta* specific gene families were also identified (Fig. 4B). The ML phylogenetic tree based on an orthologous set of 5134 single-copy coding genes indicated that *M*. *mutica* was most closely related to *T*. *scripta*, *C*. *picta* and *P*. *megacephalum*, and turtles were the sister group of crocodilians and birds, consistent with a previous investigation based on the draft genomes of *C*. *mydas* and *P*. *sinensis* (Fig. 4C)^25^. Molecular clock analysis with divergence time constraints based on fossil records revealed that lizard-snake-tuatara clade diverged from the bird-crocodilian-turtle clade at approximately 267.0–312.3 Mya, and turtles separated from the ancestor of archosaurians approximately 250.4 Mya with 95% confidence intervals between 241.4 and 265.0 Mya, and *P*. *sinensis* diverged from other TSD turtles approximately 172.4 Mya (124.4–221.2 Mya), while *M*. *mutica* divided from *T*. *scripta*, *C*. *picta* and *P*. *megacephalum* approximately 79.3 Mya (70.9–88.7 Mya) (Fig. 4C).

### Positive selection analysis involved in sex control

**Figure 5:**
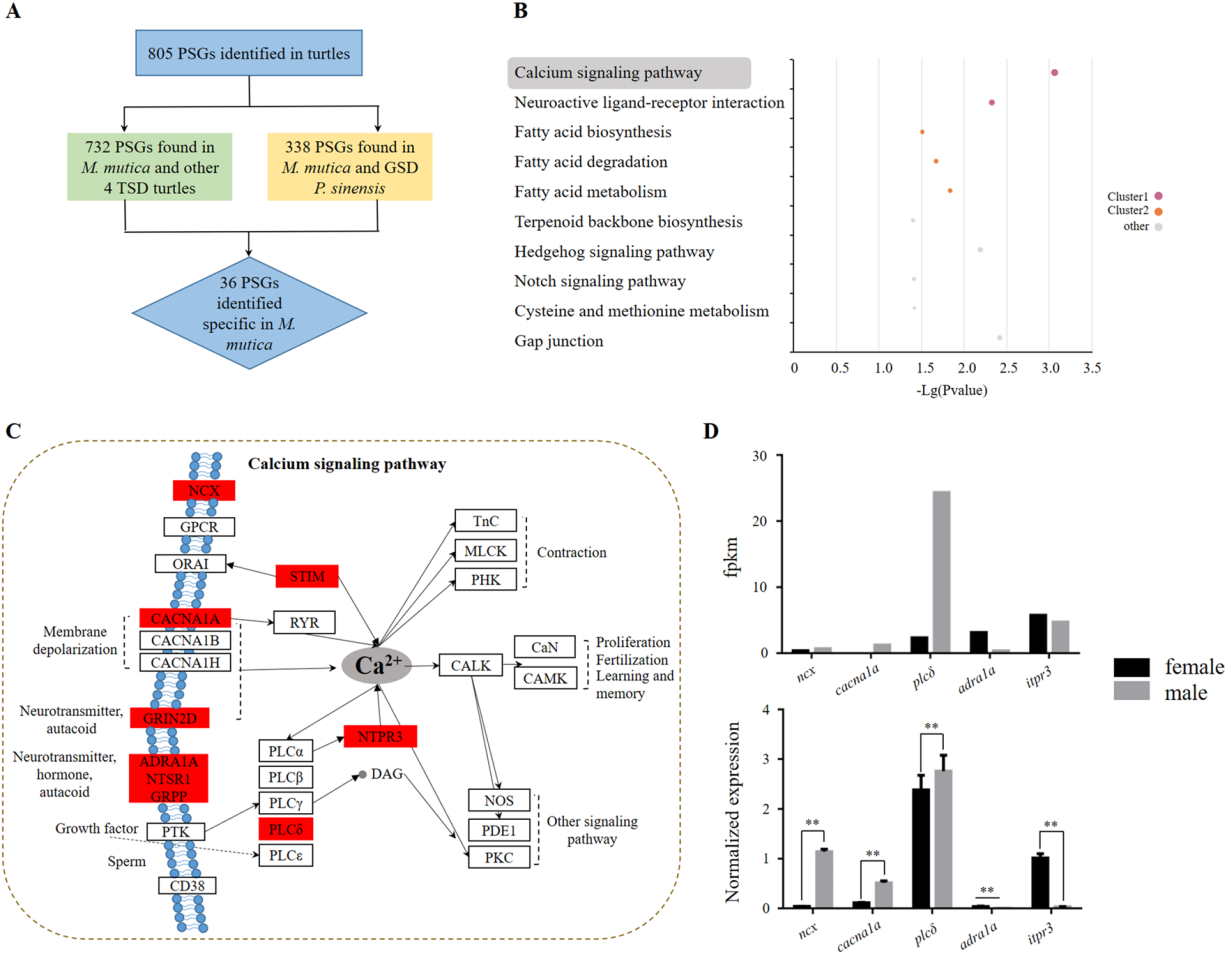
Positively selected genes and their enriched KEGG pathways in Asian yellow pond turtles. (**A**) Number of positively selected genes identified in 6 turtles (5 temperature-dependent sex determination turtles, *C*. *picta*, *T. scripta*, *C*. *mydas*, *P*. *megacephalum*, *M. mutica*, and one genotypic sex determination, *P. sinensis*). (**B**) Top 10 enriched KEGG signaling pathways of 455 TSD turtle-specific positively selected genes. (**C**) Calcium signaling pathway. The genes in the red box indicate those were positively selected. (**D**) Expression levels of positive selection genes in the calcium signaling pathway between testis and ovaries of *M. mutica*. The black and gray boxes represent the expression level in the testis and ovaries, respectively. *, *p* < 0.05; **, *p* < 0.01.

To further elucidate the potential genetic basis of sexual dimorphism and gonad development, we examined the single-copy orthologs of six turtles (*M. mutica*, *T*. *scripta*, *C. mydas*, *C. picta*, *P. megacephalum* and *P. sinensis*) to detect some key pathways or genes under positive selection using PAML software. A total of 805 PSGs (with Ka/Ks > 1) were identified in the *M. mutica* genome, among which 732 PSGs occurred in *M. mutica* and the other 4 TSD turtles, and 338 PSGs were shared between *M. mutica* and GSD *P. sinensis* (Fig. 5A). Then, the 455 TSD turtle-specific PSGs were used for enrichment analysis in KEGG pathways, mapping to 104 pathways (Table S15). The pathway with most PSGs was calcium signaling pathway, followed by neuroactive ligand-receptor interaction, fatty acid biosynthesis, and so on (Fig. 5B and Table S15). In the most significantly enriched calcium signaling pathway, nine genes were under positive selection such as Na^+^/Ca^2+^ exchanger (*ncx*), voltage-dependent P/Q-type calcium channel subunit alpha-1A (*cacna1a*), phospholipase C delta (*plcδ*), neurotensin receptor type 1 (*ntsr1*), alpha-1A adrenergic receptor (*adra1a*) and inositol 1,4,5-trisphosphate receptor type 3 (*itpr3*) (Fig. 5C and Table S15). Based on the transcriptome data of adult gonads^29^, we found out that some of these PSGs displayed sex-biased expression that *ncx*, *cacna1a* and *plcδ* had higher expression in testis than in ovary while *adra1a* and *itpr3* had higher expression in ovary than in testis (Fig. 5D). The qRT-PCR analysis revealed that the trend of these genes expression between ovary and testis were consistent with the transcriptome data (Fig. 5D). Recently, studies on *T*. *scripta* have showed that a temperature-sensitive Ca^2+^ influx promotes phosphorylation of STAT3 (signal transducer and activator of transcription 3) and then pSTAT3 represses *Kdm6b* transcription, which blocks the male development^23^. Thus, genes from calcium signaling pathway and neuroactive ligand-receptor interaction under positive selection may be associated with TSD in *M. mutica*.

## Discussion

In this study, we generate the chromosome-level genome assembly of *M. mutica* by combining the continuous long-read, Illumina, and Hi-C technologies. The assembly yielded a high-quality reference genome with an N50 scaffold length of 141.98 Mb, which is larger than the reported turtle species *T. scripta*, *C*. *picta*, *C*. *mydas*, *P*. *megacephalum* and *P*. *sinensis* (Table S4 and Table 1). Among these genome sequences represented in the NCBI genome database, only the genomes of *T. scripta* and *M. mutica* are assembled to the chromosome level, which provide valuable resources for further clarifying and exploring genomic innovations and phylogenetic origin of *M. mutica*.

Turtles have piqued researchers’ interest for a long time, as said by Shaffer et al. ^24,73^, ‘the chelonians are the most bizarre, and yet in many respects the most conservative, of reptilian groups. Because they are still living, turtles are commonplace objects to us; were they entirely extinct, they would be a cause for wonder’. It was known that turtles contain three hypotheses to their evolutionary origins: (1) they are members of early-diverged reptiles, called anapsids^73^; (2) they are closely related to the lizard-snake-tuatara (Lepidosauria) lineage^74^; and (3) they form a sister group of the crocodilians and birds (Archosauria) lineage^25,75,76^. With the advancement of multiple biotechnologies and molecular markers, the first two hypotheses have been ruled out. Here, our genome-wide phylogenetic analysis of the list turtle species also robustly confirms a close relationship of turtles to the bird-crocodilian lineage (Fig. 4C). The molecular clock of the time-calibrated phylogeny based on fossil records indicated that the divergence time between turtles and the ancestor of archosaurians was consistent with previous investigations on the origin of shells and the unique body plan of turtles^25,77^. Based on previous known cytogenetic data on chromosome numbers and Hi-C analysis, 12 microchromosome-pairs were identified beside 14 macrochromosome-pairs in *M. mutica* genome (Fig. 2). Previous work at the cytological level implied that most birds have extremely conserved karyotypes, including 9 pairs of macrochromosomes and 30–32 pairs of microchromosomes^71^. While, turtles and snakes have fewer microchromosomes than birds^78,79^. Recent studies on the origin and fate of microchromosomes in genomes of reptiles, birds, and mammals revealed that microchromosomes retain a high frequency of interchromosome interaction inside thenucleus and regularly locate together at interphase and division^80^.

Moreover, we detected several pathways related to temperature sensing/transducing and sex determination/differentiation, such as the calcium signaling pathway, neuroactive ligand-receptor interaction, oocyte meiosis, progesterone-mediated oocyte maturation and steroid hormone biosynthesis, based on selective pressure analyses (Table S15). Some key functional genes involved in the top 2 significant enrichment pathways, calcium signaling pathway and neuroactive ligand-receptor interaction were positively selected (Fig. 5). Multiple hormone-related genes in neuroactive ligand-receptor interaction pathway play a vital role in mammalian reproduction^81,82^. Moreover, five PSGs, *ncx*, *cacna1a*, *plcδ*, *adra1a* and *itpr3* in the KEGG pathway “calcium signaling” with functions involved in sexual dimorphism have been elucidated in diverse species, such as ascidians^83^, humans^84^ and rats^85^. For example, in ascidians, *ncx* has been revealed to play significant roles in the regulation of sperm-activating and sperm-attracting factor-induced sperm chemotaxis, motility activation and motility maintenance^83^. The prime activation target of Ca^2+^-calmodulin expressed in granulosa-luteal cells of swine can drive the in vitro transcriptional activity of the CYP11A promoter^86^. In closely related species of *M. mutica*, the influx of intracellular Ca^2+^ and increased reactive oxygen species levels could act as a temperature-sensitive factor to activate the pSTAT3-Kdm6b loop to stabilize ovary or testis development^23^ in *T. scripta*. Moreover, intracellular calcium ion concentration ([Ca2+]i) also plays significant tool in regulating the dynamics of GnRH neuron burst firing^87^. In our investigation, combined transcriptome analysis indicated that these PSGs also showed differential expression patterns between testes and ovaries in *M. mutica* (Fig. 5C), suggesting their potential roles in the sexual development or the maintenance or function of the testis versus ovary of *M. mutica*.

## Conclusion

In this study, we present a chromosomal-scale genome assembly of *M. mutica* using continuous long-read, Illumina, and Hi-C technologies, acquiring a total size of 2.23 Gb, with contig N50 of 8.53 Mb and scaffold N50 of 141.98 Mb. This scaffold N50 is the highest among all currently sequenced turtle genomes. Genome Hi-C scaffolding resulted in 26 pseudochromosomes containing 99.98% of the total assembly. Comparative genomics analysis indicated that the lizard-snake-tuatara clade diverged from the bird-crocodilian-turtle clade at approximately 267.0–312.3 Mya. Moreover, many genes under positive selection are from calcium signaling pathway and neuroactive ligand-receptor interaction that are involved in the process of temperature-dependent sex determination, providing important evolutionary insights into temperature-dependent sex determination system.

## Supplementary Information

Supplementary Information.

## Data Availability

Chromosome-level data of *Mauremys mutica* genome are deposited at NCBI Sequence Read Archive database under the BioSample accession number of SRR14883730 (BioProject ID: PRJNA740058). The detailed information of the raw data was shown in the tables below.
