# Supplementary material for: Chromosome-level genome assembly of Asian yellow pond turtle (Mauremys mutica) with temperature-dependent sex determination system
Source: Sci Rep. 2022 May 12;12:7905. doi: 10.1038/s41598-022-12054-2 (PMC9098631; doi:10.1038/s41598-022-12054-2)
Supplement: Supplementary file 1 — Supplementary Information. [file 41598_2022_12054_MOESM1_ESM.docx]

**Supplemental Information for:**

# Chromosome-level genome assembly of Asian yellow pond turtle (*Mauremys mutica*) with temperature-dependent sex determination system

Xiaoli Liu^1^, Yakun Wang^1^, Ju Yuan^1, 2^, Fang Liu^1^, Xiaoyou Hong^1^, Lingyun Yu^1^, Wei Li^1^, Chen Chen^1^, Wei Ni^1, 2^, Haiyang Liu^1^, Jian Zhao^1^, Chengqing Wei^1^, Haigang Chen^1^, Yihui Liu^1^, Xinping Zhu^1*^

^1^Key Laboratory of Tropical & Subtropical Fishery Resource Application & Cultivation of Ministry of Agriculture and Rural Affairs, Pearl River Fisheries Research Institute, Chinese Academy of Fishery Sciences, Guangzhou, 51038

^2^ College of Life Science and Fisheries, Shanghai Ocean University, Shanghai, China, 201306

**Contents:**

| **Supplemental_Fig_S1** | Page 3 |
| --- | --- |
| **Supplemental_Fig_S2** | Page 4 |
| **Supplemental_Table S1** | Page 4 |
| **Supplemental_Table S2** | Page 5 |
| **Supplemental_Table S3** | Page 5 |
| **Supplemental_Table S4** | Page 5 |
| **Supplemental_Table S5** | Page 5 |
| **Supplemental_Table S6** | Page 6 |
| **Supplemental_Table S7** | Page 6 |
| **Supplemental_Table S8** | Page 6 |
| **Supplemental_Table S9** | Page 7 |
| **Supplemental_Table S10** | Page 7 |
| **Supplemental_Table S11** | Page 7 |
| **Supplemental_Table S12** | Page 12 |
| **Supplemental_Table S13** | Page 12 |
| **Supplemental_Table S14** | Page 12 |
| **Supplemental_Table S15** | Page 13 |

[**Supplemental_files** 2](#_Toc45181688)

Supplemental_Fig_S1. A 21-mer distribution of the Illumina short reads to estimate genome size, ratio of repeat sequences and heterogeneity. The x-axis represents the sequencing depth of each unique 21-mer, and the y-axis represents the frequency of unique 21-mers. The k-mer depth value of the main peak is 53. After removing data with abnormal depth, a total of 142,693,993,736 21-mers were used to further estimate the genome size; thus, we first estimated that the genome size of *M. mutica* was 2.69 Gb. 3

[Supplemental_Fig_S2. Venn diagram representation of the functional annotation from different public databases, including Kyoto Encyclopedia of Genes and Genomes (KEGG) (Kanehisa & Goto, 2000), KOG (clusters of orthologous groups for eukaryotic complete genomes) (Tatusov et al., 2003), TrEMBL (Boeckmann et al., 2003), NCBI nonredundant protein sequences (NR) and GO (Gene Ontology).](#_Toc45181690) 4

[Supplemental_Table S1. The primer pairs used for RT-PCR in this study... 4](#_Toc45181693)

[Supplemental_Table S2. Statistics of *k*-mer analysis and heterozygosity in Asian yellow pond turtles.. 5](#_Toc45181693)

[Supplemental_Table S3. Statistics of short-read Illumina in the Asian yellow pond turtle genomic survey. 5](#_Toc45181694)

[Supplemental_Table S4. Statistics of Hi-C-assisted assembly of the Asian yellow pond turtle. 5](#_Toc45181695)

[Supplemental_Table S5. Results of ordering and orienting the 26 chromosome-scale pseudomolecules (pseudochromosomes, pchrs) in the Asian yellow pond turtle assembly. 5](#_Toc45181696)

[Supplemental_Table S6. Alignment of the Illumina reads to the *Mauremys mutica* genome assembly. 6](#_Toc45181697)

[Supplemental_Table S7. CEGMA assessment of the *Mauremys mutica* genome assembly. 6](#_Toc45181698)

[Supplemental_Table S8. Quality assessment of the genome using the vertebrata_odb9 database.. 6](#_Toc45181693)

[Supplemental_Table S9. Homology search against the Repbase database. 7](#_Toc45181694)

[Supplemental_Table S10. Gene family cluster statistical information. 7](#_Toc45181695)

[Supplemental_Table S11. 176 species-specific orthogroups identified in the Asian yellow pond turtle. 7](#_Toc45181696)

[Supplemental_Table S12. Length distribution of genes, CDSs, exons and introns in Asian yellow pond turtles.. 12](#_Toc45181697)

[Supplemental_Table S13. Functional annotation from the genome assembly of the Asian yellow pond turtle. 12](#_Toc45181698)

[Supplemental_Table S14. Predicted nonprotein coding genes in the Asian yellow pond turtle. 12](#_Toc45181693)

[Supplemental_Table S15. Enriched KEGG pathways of positively selected genes in *M. mutica*. 1](#_Toc45181693)3

# Supplemental_Figure


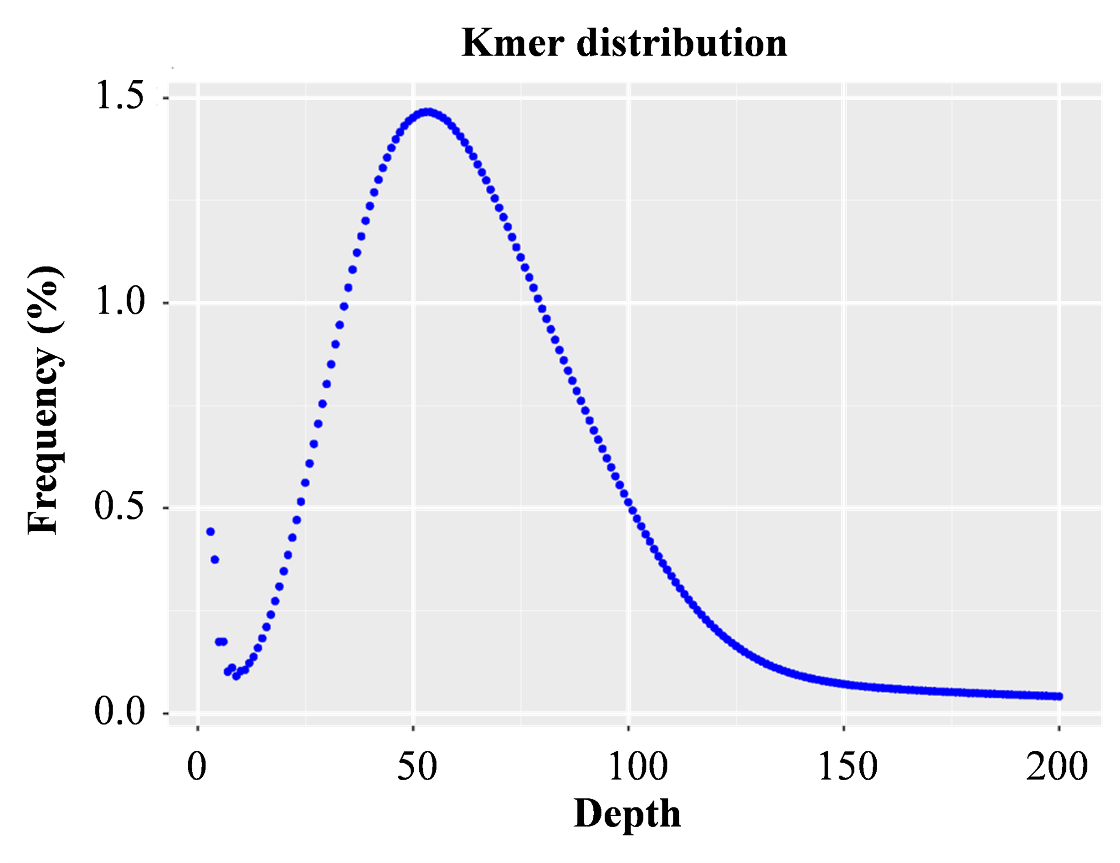


**Supplemental_Fig_S1.** A 21-mer distribution of the Illumina short reads to estimate genome size, ratio of repeat sequences and heterogeneity. The x-axis represents the sequencing depth of each unique 21-mer, and the y-axis represents the frequency of unique 21-mers. The *k*-mer depth value of the main peak is 53. After removing data with abnormal depth, a total of 142,693,993,736 21-mers were used to further estimate the genome size; thus, we first estimated that the genome size of *M*. *mutica* was 2.69 Gb.

**
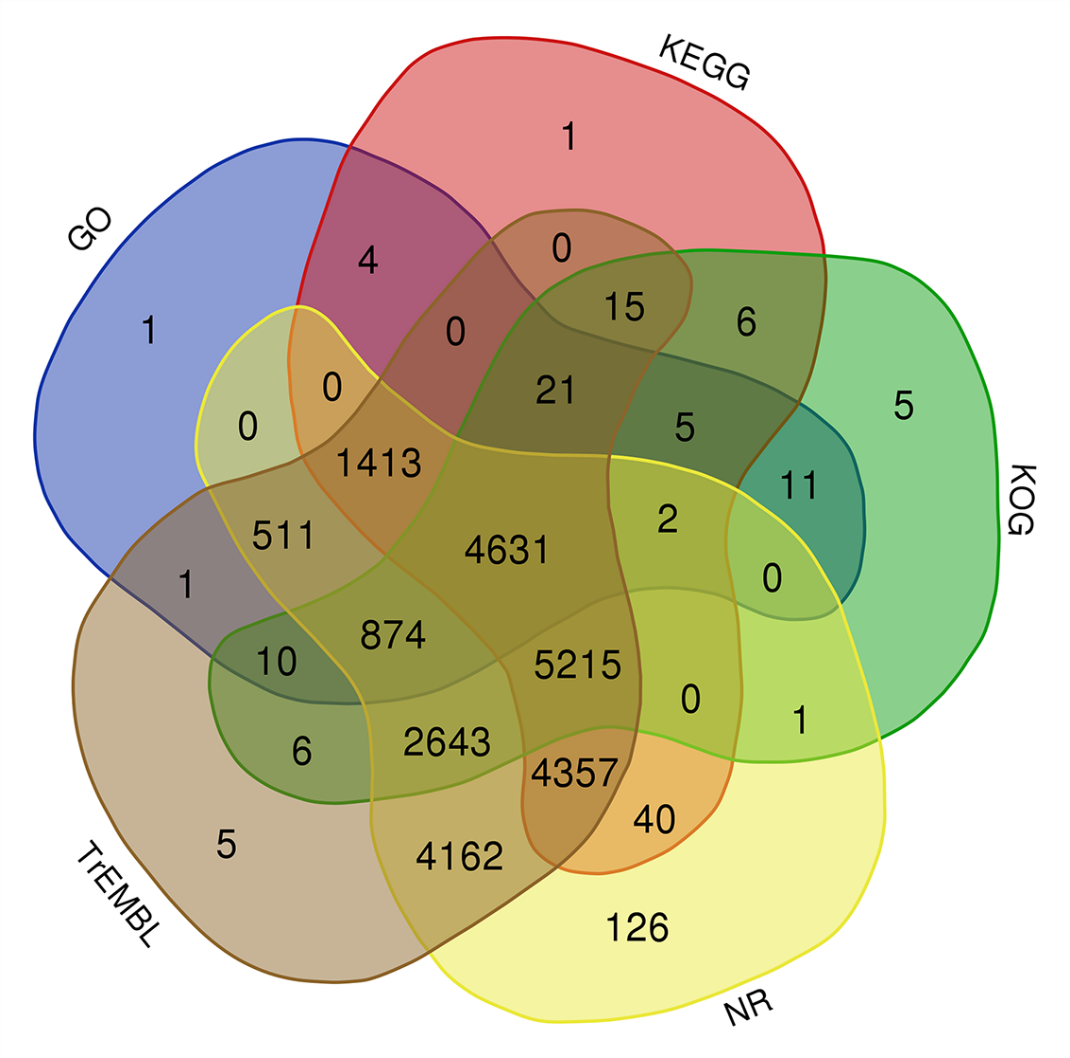
**

## Supplemental_Fig_S2. Venn diagram representation of the functional annotation from different public databases, including Kyoto Encyclopedia of Genes and Genomes (KEGG) (Kanehisa & Goto, 2000), KOG (clusters of orthologous groups for eukaryotic complete genomes) (Tatusov et al., 2003), TrEMBL (Boeckmann et al., 2003), NCBI nonredundant protein sequences (NR) and GO (Gene Ontology).

# Supplemental_Tables

**Table S1.** The primer pairs used for RT-PCR in this study.

| Gene name | Primer sequence | Amplicon size (bp) |
| --- | --- | --- |
| *β-actin* | F: 5′-GTGGCTATCCAGGCTGTGCT-3′  R: 5′-TGGTGGTGAAGCTGTAGCCTC-3′ | 168 |
| *ncx* | F: 5′-TATCGCATTCAGGCTACTCGG-3′  R: 5′-TAACGGTGGCAGTGGAGGG-3′ | 498 |
| *itpr3* | F: 5′-AACTGAGGAGCAATGGAGATAA-3′  R: 5′-ATAGTTCCCAGTGGCGAGA-3′ | 440 |
| *cacna1a* | F: 5′-CGAGCAGGAGGAGAATGGG-3′  R: 5′-TGGCAATCGCTTGATGGTT-3′ | 460 |
| *adra1a* | F: 5′-ACCTCGGTACAGAGCAGAT-3′  R: 5′-CTACAGGGATACGGACAAC-3′ | 420 |
| *plcδ* | F: 5′-GTCGTCGTCGCACAATACC-3′  R: 5′-TATGCCACTGTTCCCGTTC-3′ | 450 |

# Table S2. Statistics of *k*-mer analysis and heterozygosity in Asian yellow pond turtles.

| *k*-mer | *k*-mer depth | *k*-mer number | filtered *k*-mer number | Genome size(bp) | Heterozygosity  (%) | Repeat sequence content (%) |
| --- | --- | --- | --- | --- | --- | --- |
| 21 | 53 | 148,757,912,763 | 142,693,993,736 | 2,692,339,504 | 0.60 | 52.10 |

# Table S3. Statistics of short-read Illumina in the Asian yellow pond turtle genomic survey.

| Reads Type | Reads Num | Total Bases(bp) | Reads N50 (bp) | Reads mean Length(bp) | Longest Read (bp) |
| --- | --- | --- | --- | --- | --- |
| Subreads | 15,644,350 | 280,424,529,332 | 26,874 | 17,925 | 309,763 |

# Table S4. Statistics of Hi-C-assisted assembly of the Asian yellow pond turtle.

|  | Total (bp) | Contig Number | Contigs  N50 (bp) | Contig Length Proportion | Contig Number (>100kb) | Contig Length(>100kb) | Contig Length Proportion(>100kb) | Scafflod Number | Scafflod  N50 (bp) | GC Content  (%) |
| --- | --- | --- | --- | --- | --- | --- | --- | --- | --- | --- |
| Before the assembly using Hi-C | 2,361,558,986 | 1,530 | 8,609,801 | - | 1,097 | 2,330,098,296 | - | - | - | 45.11 |
| After the assembly using Hi-C | 2,228,770,873 | 1,106 | 8,525,479 | 94.38 | 717 | 2,211,083,089 | 94.89% | 26 | 141,975,148 | - |

# Table S5. Results of ordering and orienting the 26 chromosome-scale pseudomolecules (pseudochromosomes, pchrs) in the Asian yellow pond turtle assembly.

| Pseudo-chromosome | Number of Contigs | Length of Contigs | Length of Pseudo-chromosome (bp) |
| --- | --- | --- | --- |
| Pseudo-chromosome1 | 155 | 368,402,959 | 368,479,959 |
| Pseudo-chromosome2 | 117 | 296,401,046 | 296,459,046 |
| Pseudo-chromosome3 | 53 | 210,384,463 | 210,410,463 |
| Pseudo-chromosome4 | 86 | 155,036,731 | 155,079,231 |
| Pseudo-chromosome5 | 34 | 141,958,648 | 141,975,148 |
| Pseudo-chromosome6 | 27 | 131,567,000 | 131,580,000 |
| Pseudo-chromosome7 | 37 | 129,514,811 | 129,532,811 |
| Pseudo-chromosome8 | 28 | 111,775,452 | 111,788,952 |
| Pseudo-chromosome9 | 17 | 105,792,839 | 105,800,839 |
| Pseudo-chromosome10 | 36 | 86,823,000 | 86,840,500 |
| Pseudo-chromosome11 | 89 | 80,739,745 | 80,783,745 |
| Pseudo-chromosome12 | 114 | 59,457,636 | 59,514,136 |
| Pseudo-chromosome13 | 10 | 43,530,068 | 43,534,568 |
| Pseudo-chromosome14 | 15 | 33,647,547 | 33,654,547 |
| Pseudo-chromosome15 | 16 | 33,377,978 | 33,385,478 |
| Pseudo-chromosome16 | 64 | 30,039,250 | 30,070,750 |
| Pseudo-chromosome17 | 4 | 25,878,678 | 25,880,178 |
| Pseudo-chromosome18 | 16 | 25,622,785 | 25,630,285 |
| Pseudo-chromosome19 | 45 | 24,257,807 | 24,279,807 |
| Pseudo-chromosome20 | 26 | 23,797,105 | 23,809,605 |
| Pseudo-chromosome21 | 36 | 21,301,010 | 21,318,510 |
| Pseudo-chromosome22 | 6 | 19,538,033 | 19,540,533 |
| Pseudo-chromosome23 | 7 | 19,142,902 | 19,145,902 |
| Pseudo-chromosome24 | 45 | 17,497,420 | 17,519,420 |
| Pseudo-chromosome25 | 11 | 16,975,839 | 16,980,839 |
| Pseudo-chromosome26 | 12 | 16,310,121 | 16,315,621 |
| Total | 1,106 | 2,228,770,873 | 2,229,310,873 |

# Table S6. Alignment of the Illumina reads to the *Mauremys mutica* genome assembly.

| Total_reads | Mapped_reads | Mapped(%) | Properly_mapped_reads | Properly_mapped(%) |
| --- | --- | --- | --- | --- |
| 1,162,168,915 | 1,158,518,153 | 99.69 | 1,127,909,063 | 97.05 |

# Table S7. CEGMA assessment of the *Mauremys mutica* genome assembly.

| Number of 458 CEG present in assembly | %of 458 CEGs present in assemblies | Number of 248 highly conserved CEGs present | % of 248 highly conserved CEGs present |
| --- | --- | --- | --- |
| 449 | 98.03% | 231 | 93.15% |

# Table S8. Quality assessment of the genome using the vertebrata_odb9 database.

| Term | BUSCO number | Proportion(%) |
| --- | --- | --- |
| Complete BUSCOs | 2,494 | 96.44 |
| Complete and single-copy BUSCOs | 2,462 | 95.20 |
| Complete and duplicated BUSCOs | 32 | 1.24 |
| Fragmented BUSCOs | 61 | 2.36 |
| Missing BUSCOs | 31 | 1.20 |
| Total BUSCO groups searched | 2,586 | 100.00 |

# Table S9. Homology search against the Repbase database.

| Type | Number | Length(bp) | Rate(%) |
| --- | --- | --- | --- |
| ClassI/DIRS | 139,940 | 63,771,922 | 2.7 |
| ClassI/LARD | 733,385 | 136,965,769 | 5.8 |
| ClassI/LINE | 1,545,889 | 398,499,014 | 16.87 |
| ClassI/LTR/Copia | 407,416 | 163,680,585 | 6.92 |
| ClassI/PLE | 1,478,286 | 307,646,242 | 13.02 |
| ClassI/SINE | 21,535 | 3,752,596 | 0.16 |
| ClassI/TRIM | 1,964 | 1,086,924 | 0.05 |
| ClassII/Crypton | 705 | 435,804 | 0.02 |
| ClassII/Helitron | 3,186 | 371,699 | 0.02 |
| ClassII/MITE | 4,554 | 669,621 | 0.03 |
| ClassII/Maverick | 10,709 | 3,671,593 | 0.16 |
| ClassII/TIR | 1,204,722 | 309,668,610 | 13.11 |
| PotentialHostGene | 8,517 | 3,558,324 | 0.15 |
| SSR | 1,192 | 260,322 | 0.01 |
| Unknown | 322,909 | 54,550,086 | 2.31 |
| Total | 5,884,909 | 1,448,589,111 | 61.33 |

# Table S10. Gene family cluster statistical information.

|  | *M. mutica* | *A. carolinensis* | *A*. *mississippiensis* | *C. mydas* | *C. picta* | D. acutus | *G*. *gallus* | *H*. *sapiens* | *M*. *musculus* | *P*. *megacephalum* | *P*. *sinensis* |
| --- | --- | --- | --- | --- | --- | --- | --- | --- | --- | --- | --- |
| Number of genes | 24,751 | 18,591 | 18,931 | 18,046 | 21,177 | 20,743 | 17,397 | 23,023 | 24,018 | 21,529 | 19,380 |
| Number of genes in orthogroups | 22,593 | 16,255 | 17,819 | 16,899 | 20,250 | 19,306 | 16,139 | 21,059 | 22,030 | 16,613 | 17,955 |
| Number of unassigned genes | 2,158 | 2,336 | 1,112 | 1,147 | 927 | 1,437 | 1,258 | 1,964 | 1,988 | 4,916 | 1,425 |
| Percentage of genes in orthogroups | 91.3 | 87.4 | 94.1 | 93.6 | 95.6 | 93.1 | 92.8 | 91.5 | 91.7 | 77.2 | 92.6 |
| Percentage of unassigned genes | 8.7 | 12.6 | 5.9 | 6.4 | 4.4 | 6.9 | 7.2 | 8.5 | 8.3 | 22.8 | 7.4 |
| Number of orthogroups containing species | 18,126 | 14,599 | 16,424 | 15,565 | 17,584 | 15,373 | 14,386 | 17,263 | 17,556 | 15,596 | 15,443 |
| Percentage of orthogroups containing species | 67.6 | 54.4 | 61.3 | 58.0 | 65.6 | 57.3 | 53.7 | 64.4 | 65.5 | 58.2 | 57.6 |
| Number of species-specific orthogroups | 176 | 138 | 57 | 17 | 47 | 245 | 165 | 517 | 684 | 102 | 68 |
| Number of genes in species-specific orthogroups | 490 | 508 | 152 | 35 | 103 | 2,188 | 1,028 | 2,095 | 3,373 | 221 | 209 |
| Percentage of genes in species-specific orthogroups | 2.0 | 2.7 | 0.8 | 0.2 | 0.5 | 10.5 | 5.9 | 9.1 | 14.0 | 1.0 | 1.1 |

# Table S11. 176 species-specific orthogroups identified in the Asian yellow pond turtle.

|  | Orthogroups_Single Copy Orthologues | Orthogroups_Specific_Gene_Family |
| --- | --- | --- |
| 1 | OG0007707 | EVM0003889,EVM0006179,EVM0011186,EVM0014363,EVM0018415,EVM0020633,EVM0021556,EVM0022477,EVM0022862,EVM0022951,EVM0023231,EVM0023583 |
| 2 | OG0010733 | EVM0000564,EVM0000925,EVM0002152,EVM0008851,EVM0009020,EVM0011019,EVM0011149,EVM0011424,EVM0012271,EVM0015004,EVM0018696 |
| 3 | OG0010734 | EVM0003031,EVM0003123,EVM0003328,EVM0003555,EVM0004087,EVM0008151,EVM0014703,EVM0019777,EVM0020887,EVM0022438,EVM0023114 |
| 4 | OG0016001 | EVM0006838,EVM0006846,EVM0015844,EVM0018862,EVM0021486,EVM0023563,EVM0023871 |
| 5 | OG0017512 | EVM0000202,EVM0006718,EVM0007857,EVM0017387,EVM0018781,EVM0021399 |
| 6 | OG0017514 | EVM0006077,EVM0009223,EVM0017447,EVM0019300,EVM0020320,EVM0020867 |
| 7 | OG0017516 | EVM0001891,EVM0008316,EVM0008823,EVM0015672,EVM0018499,EVM0024685 |
| 8 | OG0017518 | EVM0000642,EVM0003349,EVM0006809,EVM0010744,EVM0016098,EVM0021764 |
| 9 | OG0019032 | EVM0006502,EVM0015347,EVM0019996,EVM0022653,EVM0023901 |
| 10 | OG0019034 | EVM0000820,EVM0004716,EVM0010499,EVM0015744,EVM0017734 |
| 11 | OG0019037 | EVM0005462,EVM0005624,EVM0012020,EVM0015307,EVM0017827 |
| 12 | OG0019042 | EVM0000457,EVM0015259,EVM0020870,EVM0021800,EVM0021829 |
| 13 | OG0019043 | EVM0003466,EVM0004584,EVM0006338,EVM0021217,EVM0023375 |
| 14 | OG0019044 | EVM0002199,EVM0005267,EVM0010670,EVM0015459,EVM0023052 |
| 15 | OG0019046 | EVM0003325,EVM0004447,EVM0008780,EVM0017379,EVM0022010 |
| 16 | OG0019047 | EVM0002062,EVM0002319,EVM0004009,EVM0020456,EVM0021439 |
| 17 | OG0019048 | EVM0002615,EVM0003807,EVM0008620,EVM0011014,EVM0019519 |
| 18 | OG0020558 | EVM0008435,EVM0011724,EVM0017619,EVM0018626 |
| 19 | OG0020559 | EVM0011251,EVM0017621,EVM0019871,EVM0024036 |
| 20 | OG0020568 | EVM0005316,EVM0005983,EVM0009721,EVM0012004 |
| 21 | OG0020571 | EVM0009243,EVM0012780,EVM0016783,EVM0018718 |
| 22 | OG0020573 | EVM0000631,EVM0003863,EVM0009677,EVM0023855 |
| 23 | OG0020574 | EVM0001354,EVM0006909,EVM0007343,EVM0018786 |
| 24 | OG0020578 | EVM0000395,EVM0006514,EVM0011093,EVM0020987 |
| 25 | OG0020580 | EVM0002623,EVM0005044,EVM0007212,EVM0009088 |
| 26 | OG0020583 | EVM0007361,EVM0011456,EVM0011994,EVM0019751 |
| 27 | OG0020585 | EVM0009649,EVM0012063,EVM0015303,EVM0018818 |
| 28 | OG0020590 | EVM0015528,EVM0017989,EVM0021470,EVM0022494 |
| 29 | OG0022338 | EVM0010424,EVM0017476,EVM0020631 |
| 30 | OG0022339 | EVM0001639,EVM0005282,EVM0012254 |
| 31 | OG0022340 | EVM0000540,EVM0014800,EVM0016589 |
| 32 | OG0022341 | EVM0006600,EVM0006690,EVM0008652 |
| 33 | OG0022342 | EVM0018737,EVM0018778,EVM0022657 |
| 34 | OG0022345 | EVM0003843,EVM0022016,EVM0024343 |
| 35 | OG0022346 | EVM0011868,EVM0014124,EVM0018989 |
| 36 | OG0022347 | EVM0014214,EVM0018164,EVM0021135 |
| 37 | OG0022348 | EVM0004780,EVM0013851,EVM0021213 |
| 38 | OG0022349 | EVM0014379,EVM0014936,EVM0024451 |
| 39 | OG0022354 | EVM0000162,EVM0006102,EVM0024353 |
| 40 | OG0022356 | EVM0004988,EVM0009808,EVM0015088 |
| 41 | OG0022357 | EVM0013801,EVM0019845,EVM0020267 |
| 42 | OG0022358 | EVM0000066,EVM0009847,EVM0022404 |
| 43 | OG0022359 | EVM0006387,EVM0013095,EVM0018147 |
| 44 | OG0022360 | EVM0007242,EVM0017773,EVM0023684 |
| 45 | OG0022361 | EVM0003951,EVM0007008,EVM0008929 |
| 46 | OG0022363 | EVM0000255,EVM0012146,EVM0024512 |
| 47 | OG0022364 | EVM0010314,EVM0011874,EVM0023398 |
| 48 | OG0022365 | EVM0005692,EVM0006419,EVM0020605 |
| 49 | OG0022366 | EVM0012873,EVM0020337,EVM0022615 |
| 50 | OG0022371 | EVM0001584,EVM0014423,EVM0021750 |
| 51 | OG0022372 | EVM0004251,EVM0009134,EVM0017276 |
| 52 | OG0022374 | EVM0008975,EVM0009930,EVM0019039 |
| 53 | OG0022380 | EVM0008410,EVM0012583,EVM0023108 |
| 54 | OG0022382 | EVM0002078,EVM0002729,EVM0006777 |
| 55 | OG0022383 | EVM0009764,EVM0014226,EVM0023291 |
| 56 | OG0022386 | EVM0001446,EVM0003145,EVM0010458 |
| 57 | OG0022388 | EVM0009497,EVM0009884,EVM0012386 |
| 58 | OG0022389 | EVM0002422,EVM0009747,EVM0016303 |
| 59 | OG0022391 | EVM0010953,EVM0013954,EVM0018517 |
| 60 | OG0022395 | EVM0001157,EVM0009043,EVM0023135 |
| 61 | OG0022397 | EVM0004961,EVM0010128,EVM0011244 |
| 62 | OG0022400 | EVM0002841,EVM0009478,EVM0024029 |
| 63 | OG0022401 | EVM0011004,EVM0011682,EVM0015940 |
| 64 | OG0022407 | EVM0007235,EVM0008724,EVM0011644 |
| 65 | OG0022412 | EVM0000996,EVM0015157,EVM0023778 |
| 66 | OG0022418 | EVM0002304,EVM0002734,EVM0012949 |
| 67 | OG0022426 | EVM0004390,EVM0004992,EVM0021235 |
| 68 | OG0022432 | EVM0001316,EVM0015452,EVM0021406 |
| 69 | OG0026149 | EVM0005694,EVM0008534 |
| 70 | OG0026152 | EVM0000660,EVM0023978 |
| 71 | OG0026153 | EVM0009464,EVM0014020 |
| 72 | OG0026155 | EVM0005315,EVM0011295 |
| 73 | OG0026157 | EVM0003471,EVM0009761 |
| 74 | OG0026161 | EVM0007202,EVM0018116 |
| 75 | OG0026164 | EVM0006802,EVM0014766 |
| 76 | OG0026166 | EVM0004286,EVM0007102 |
| 77 | OG0026168 | EVM0001920,EVM0024086 |
| 78 | OG0026170 | EVM0000443,EVM0018040 |
| 79 | OG0026178 | EVM0000765,EVM0004502 |
| 80 | OG0026185 | EVM0003270,EVM0003982 |
| 81 | OG0026187 | EVM0007338,EVM0015142 |
| 82 | OG0026188 | EVM0004213,EVM0009034 |
| 83 | OG0026191 | EVM0010457,EVM0022264 |
| 84 | OG0026198 | EVM0002730,EVM0023696 |
| 85 | OG0026199 | EVM0004010,EVM0005241 |
| 86 | OG0026205 | EVM0003097,EVM0008260 |
| 87 | OG0026207 | EVM0013371,EVM0017732 |
| 88 | OG0026208 | EVM0002463,EVM0008571 |
| 89 | OG0026209 | EVM0006080,EVM0015312 |
| 90 | OG0026210 | EVM0015656,EVM0019581 |
| 91 | OG0026212 | EVM0020717,EVM0021446 |
| 92 | OG0026226 | EVM0010335,EVM0024665 |
| 93 | OG0026230 | EVM0007238,EVM0011515 |
| 94 | OG0026240 | EVM0012269,EVM0012560 |
| 95 | OG0026245 | EVM0013529,EVM0017611 |
| 96 | OG0026247 | EVM0001449,EVM0016603 |
| 97 | OG0026248 | EVM0003415,EVM0005041 |
| 98 | OG0026250 | EVM0009917,EVM0024136 |
| 99 | OG0026252 | EVM0003212,EVM0018319 |
| 100 | OG0026255 | EVM0011698,EVM0016967 |
| 101 | OG0026258 | EVM0007454,EVM0022498 |
| 102 | OG0026263 | EVM0009815,EVM0021646 |
| 103 | OG0026267 | EVM0002160,EVM0023012 |
| 104 | OG0026269 | EVM0011451,EVM0016196 |
| 105 | OG0026270 | EVM0010857,EVM0017793 |
| 106 | OG0026273 | EVM0009676,EVM0011705 |
| 107 | OG0026277 | EVM0021751,EVM0024131 |
| 108 | OG0026286 | EVM0010825,EVM0011916 |
| 109 | OG0026287 | EVM0005855,EVM0013747 |
| 110 | OG0026288 | EVM0002868,EVM0018188 |
| 111 | OG0026291 | EVM0011624,EVM0012810 |
| 112 | OG0026298 | EVM0015854,EVM0019352 |
| 113 | OG0026300 | EVM0008020,EVM0016587 |
| 114 | OG0026304 | EVM0017803,EVM0018270 |
| 115 | OG0026308 | EVM0014156,EVM0020916 |
| 116 | OG0026311 | EVM0002702,EVM0021431 |
| 117 | OG0026318 | EVM0000938,EVM0004182 |
| 118 | OG0026323 | EVM0005690,EVM0017134 |
| 119 | OG0026324 | EVM0010662,EVM0010725 |
| 120 | OG0026328 | EVM0011562,EVM0021473 |
| 121 | OG0026329 | EVM0000840,EVM0015305 |
| 122 | OG0026333 | EVM0013211,EVM0015463 |
| 123 | OG0026336 | EVM0009905,EVM0010692 |
| 124 | OG0026338 | EVM0014704,EVM0020412 |
| 125 | OG0026345 | EVM0012013,EVM0018784 |
| 126 | OG0026347 | EVM0018670,EVM0020423 |
| 127 | OG0026349 | EVM0000170,EVM0012879 |
| 128 | OG0026350 | EVM0015726,EVM0017021 |
| 129 | OG0026358 | EVM0014102,EVM0022387 |
| 130 | OG0026361 | EVM0005118,EVM0010869 |
| 131 | OG0026362 | EVM0002957,EVM0017259 |
| 132 | OG0026365 | EVM0007792,EVM0011083 |
| 133 | OG0026366 | EVM0013854,EVM0023041 |
| 134 | OG0026368 | EVM0001064,EVM0018726 |
| 135 | OG0026370 | EVM0001016,EVM0007690 |
| 136 | OG0026375 | EVM0001399,EVM0016367 |
| 137 | OG0026377 | EVM0012639,EVM0014554 |
| 138 | OG0026379 | EVM0011725,EVM0019182 |
| 139 | OG0026380 | EVM0006131,EVM0013339 |
| 140 | OG0026382 | EVM0013273,EVM0015493 |
| 141 | OG0026388 | EVM0003570,EVM0008199 |
| 142 | OG0026391 | EVM0012760,EVM0016206 |
| 143 | OG0026392 | EVM0010792,EVM0020553 |
| 144 | OG0026400 | EVM0001267,EVM0023900 |
| 145 | OG0026401 | EVM0000386,EVM0023412 |
| 146 | OG0026403 | EVM0015781,EVM0021870 |
| 147 | OG0026407 | EVM0000234,EVM0013847 |
| 148 | OG0026409 | EVM0004047,EVM0023470 |
| 149 | OG0026413 | EVM0000653,EVM0015172 |
| 150 | OG0026417 | EVM0009871,EVM0011022 |
| 151 | OG0026419 | EVM0008011,EVM0009859 |
| 152 | OG0026422 | EVM0005141,EVM0005424 |
| 153 | OG0026424 | EVM0013562,EVM0017402 |
| 154 | OG0026426 | EVM0004077,EVM0016079 |
| 155 | OG0026428 | EVM0004158,EVM0016755 |
| 156 | OG0026430 | EVM0006209,EVM0006882 |
| 157 | OG0026432 | EVM0017779,EVM0019272 |
| 158 | OG0026439 | EVM0003253,EVM0006287 |
| 159 | OG0026449 | EVM0006345,EVM0017924 |
| 160 | OG0026455 | EVM0011089,EVM0019243 |
| 161 | OG0026463 | EVM0008098,EVM0022508 |
| 162 | OG0026467 | EVM0012844,EVM0021330 |
| 163 | OG0026469 | EVM0021481,EVM0021640 |
| 164 | OG0026472 | EVM0016022,EVM0018586 |
| 165 | OG0026474 | EVM0005956,EVM0016008 |
| 166 | OG0026488 | EVM0013473,EVM0014028 |
| 167 | OG0026495 | EVM0013345,EVM0020711 |
| 168 | OG0026504 | EVM0004745,EVM0018544 |
| 169 | OG0026505 | EVM0001143,EVM0002214 |
| 170 | OG0026520 | EVM0004265,EVM0018287 |
| 171 | OG0026524 | EVM0005940,EVM0019653 |
| 172 | OG0026531 | EVM0007300,EVM0014662 |
| 173 | OG0026542 | EVM0006779,EVM0020515 |
| 174 | OG0026550 | EVM0017157,EVM0022658 |
| 175 | OG0026554 | EVM0002568,EVM0003913 |
| 176 | OG0026566 | EVM0001543,EVM0008570 |

# Table S12. Length distribution of genes, CDSs, exons and introns in Asian yellow pond turtles.

| GeneNum | AveGenlen | AveExonLen | AveExonNum | AveCDSlen | AveCDSNum | AveIntronLen | AveIntronnum |
| --- | --- | --- | --- | --- | --- | --- | --- |
| 24751 | 26645.17 | 2491.72 | 8.7 | 1521.64 | 8.48 | 24153.44 | 7.7 |

Note：GeneNum: gene number; AveGenlen: average gene length; AveExonLen: average exon length; AveExonNum: average exon number; AveCDSlen: average CDS length; AveCDSNum: average CDS number; AveIntronLen: average intron length; AveIntronnum: average intron number

# Table S13. Functional annotation from the genome assembly of the Asian yellow pond turtle.

| Database | Annotated number | % of gene | 100<=Protein length<300 | Protein length>=300 |
| --- | --- | --- | --- | --- |
| GO Annotation | 7,483 | 31.09% | 2,596 | 4,691 |
| KEGG Annotation | 15,748 | 65.44% | 4,178 | 11,293 |
| KOG Annotation | 15,221 | 63.25% | 4,127 | 10,845 |
| TrEMBL Annotation | 23,908 | 99.34% | 7,368 | 16,044 |
| NR Annotation | 24,058 | 99.97% | 7,468 | 16,077 |
| All Annotated | 24,066 | 100% | 7,475 | 16,078 |

# Table S14. Predicted nonprotein coding genes in the Asian yellow pond turtle.

| RNA classification | Number | Family |
| --- | --- | --- |
| miRNA | 262 | 103 |
| rRNA | 219 | 4 |
| tRNA | 8499 | 25 |

# Table S15. Enriched KEGG pathways of positively selected genes in *M. mutica*.

| #Term | ID | Input number | Background number | P-Value | Input |
| --- | --- | --- | --- | --- | --- |
| MAPK signaling pathway | cpic04010 | 15 | 326 | 5.40E-05 | TGFA\|NFATC1\|NTF3\|PDGFD\|FGF9\|RASGRF2\|CACNB2\|EFNA2\|MAP3K4\|CACNB4\|INSR\|NF1\|CACNA1A\|SRF\|RELA |
| Metabolic pathways | cpic01100 | 37 | 1552 | 0.000583 | AMPD2\|MGLL\|MBOAT2\|RBKS\|DNMT3A\|ACSBG1\|PLA2G3\|ACADM\|ACAT2\|FASN\|SPTLC3\|LPCAT1\|HSD11B2\|B4GALT6\|DNMT3B\|UCK2\|EXT2\|UAP1\|MAN2A1\|CHST8\|SUCLG1\|GALNT8\|PLCD1\|PIK3C3\|MTAP\|CNDP2\|HOGA1\|ADCY6\|HLCS\|CA4\|NAPRT\|GGT1\|ANPEP\|MGAT4B\|UAP1L1\|PDE4B\|DMGDH |
| Calcium signaling pathway | cpic04020 | 10 | 216 | 0.000898 | ADRA1A\|STIM1\|GRPR\|ADRA1D\|CACNA1A\|PLCD1\|NTSR1\|GRIN2D\|ITPR3\|GRM5 |
| Gap junction | cpic04540 | 6 | 106 | 0.003768 | ADCY6\|PDGFD\|CSNK1D\|PRKG1\|GRM5\|ITPR3 |
| Neuroactive ligand-receptor interaction | cpic04080 | 13 | 414 | 0.004733 | PTH1R\|ADRA1A\|ADRA1D\|CHRND\|DRD4\|GRIA4\|NTSR1\|VIPR2\|GRIN2D\|GRM5\|GRPR\|ADCYAP1R1\|CRHR2 |
| Vascular smooth muscle contraction | cpic04270 | 7 | 152 | 0.005327 | ADRA1A\|ADCY6\|ADRA1D\|RAMP2\|PRKG1\|PLA2G3\|ITPR3 |
| Hedgehog signaling pathway | cpic04340 | 4 | 52 | 0.006408 | SMO\|GLI3\|CSNK1D\|BOC |
| Adrenergic signaling in cardiomyocytes | cpic04261 | 7 | 161 | 0.007142 | ADRA1A\|ADCY6\|ADRA1D\|PPP2R3C\|KCNQ1\|CACNB2\|CACNB4 |
| Fatty acid metabolism | cpic01212 | 4 | 67 | 0.014541 | ACAT2\|FASN\|ACSBG1\|ACADM |
| Fatty acid degradation | cpic00071 | 3 | 42 | 0.021556 | ACAT2\|ACSBG1\|ACADM |
| C-type lectin receptor signaling pathway | cpic04625 | 5 | 124 | 0.028555 | NFATC1\|PTPN11\|RELA\|PLK3\|ITPR3 |
| Fatty acid biosynthesis | cpic00061 | 2 | 19 | 0.030987 | FASN\|ACSBG1 |
| Cysteine and methionine metabolism | cpic00270 | 3 | 53 | 0.037908 | DNMT3B\|DNMT3A\|MTAP |
| Notch signaling pathway | cpic04330 | 3 | 53 | 0.037908 | RBPJ\|NUMB\|MAML1 |
| Terpenoid backbone biosynthesis | cpic00900 | 2 | 22 | 0.039689 | ACAT2\|PDSS1 |
| Cytokine-cytokine receptor interaction | cpic04060 | 7 | 236 | 0.043308 | BMP3\|IL17B\|IL17C\|CCL19\|CD4\|OSMR\|CNTFR |
| ECM-receptor interaction | cpic04512 | 4 | 99 | 0.047891 | LAMC3\|COL6A3\|LAMC1\|SDC4 |
| Lysine degradation | cpic00310 | 3 | 61 | 0.052811 | KMT2C\|ACAT2\|BBOX1 |
| Mitophagy - animal | cpic04137 | 3 | 64 | 0.059024 | BCL2L1\|RELA\|RHOT2 |
| AGE-RAGE signaling pathway in diabetic complications | cpic04933 | 4 | 108 | 0.061489 | COL3A1\|NFATC1\|RELA\|PLCD1 |
| Glyoxylate and dicarboxylate metabolism | cpic00630 | 2 | 33 | 0.077897 | HOGA1\|ACAT2 |
| Propanoate metabolism | cpic00640 | 2 | 33 | 0.077897 | ACAT2\|SUCLG1 |
| Endocytosis | cpic04144 | 7 | 272 | 0.078142 | DNAJC6\|ACAP2\|LDLR\|DNM1\|ARFGAP2\|LDLRAP1\|ASAP2 |
| Adipocytokine signaling pathway | cpic04920 | 3 | 78 | 0.092173 | PTPN11\|RELA\|ACSBG1 |
| mRNA surveillance pathway | cpic03015 | 3 | 81 | 0.100097 | SMG5\|PPP2R3C\|HBS1L |
| Ubiquitin mediated proteolysis | cpic04120 | 4 | 140 | 0.123987 | UBE3B\|PIAS3\|UBE2C\|UBE2D1 |
| Synthesis and degradation of ketone bodies | cpic00072 | 1 | 10 | 0.136166 | ACAT2 |
| Arginine and proline metabolism | cpic00330 | 2 | 48 | 0.141389 | HOGA1\|CNDP2 |
| Amino sugar and nucleotide sugar metabolism | cpic00520 | 2 | 52 | 0.15977 | UAP1\|UAP1L1 |
| Apelin signaling pathway | cpic04371 | 4 | 157 | 0.164935 | ITPR3\|ADCY6\|PIK3C3\|HDAC5 |
| Fanconi anemia pathway | cpic03460 | 2 | 54 | 0.169118 | WDR48\|FANCM |
| Valine, leucine and isoleucine degradation | cpic00280 | 2 | 55 | 0.173827 | ACAT2\|ACADM |
| N-Glycan biosynthesis | cpic00510 | 2 | 56 | 0.178556 | MAN2A1\|MGAT4B |
| Taurine and hypotaurine metabolism | cpic00430 | 1 | 14 | 0.180957 | GGT1 |
| Phosphatidylinositol signaling system | cpic04070 | 3 | 109 | 0.185006 | ITPR3\|PIK3C3\|PLCD1 |
| GnRH signaling pathway | cpic04912 | 3 | 112 | 0.195025 | ITPR3\|ADCY6\|MAP3K4 |
| NOD-like receptor signaling pathway | cpic04621 | 4 | 169 | 0.19637 | BCL2L1\|ITPR3\|TYK2\|RELA |
| Ether lipid metabolism | cpic00565 | 2 | 62 | 0.207299 | PLA2G3\|LPCAT1 |
| Sphingolipid metabolism | cpic00600 | 2 | 62 | 0.207299 | SPTLC3\|B4GALT6 |
| Cellular senescence | cpic04218 | 4 | 174 | 0.209978 | NFATC1\|E2F5\|RELA\|ITPR3 |
| RIG-I-like receptor signaling pathway | cpic04622 | 2 | 64 | 0.21699 | RELA\|TBKBP1 |
| Glycerolipid metabolism | cpic00561 | 2 | 65 | 0.22185 | MBOAT2\|MGLL |
| Arachidonic acid metabolism | cpic00590 | 2 | 65 | 0.22185 | GGT1\|PLA2G3 |
| Glutathione metabolism | cpic00480 | 2 | 65 | 0.22185 | GGT1\|ANPEP |
| Nitrogen metabolism | cpic00910 | 1 | 18 | 0.223433 | CA4 |
| Glycerophospholipid metabolism | cpic00564 | 3 | 126 | 0.243306 | MBOAT2\|PLA2G3\|LPCAT1 |
| Glycosaminoglycan biosynthesis - chondroitin sulfate / dermatan sulfate | cpic00532 | 1 | 20 | 0.24384 | CHST11 |
| Histidine metabolism | cpic00340 | 1 | 22 | 0.263713 | CNDP2 |
| p53 signaling pathway | cpic04115 | 2 | 74 | 0.265862 | BCL2L1\|STEAP3 |
| Protein export | cpic03060 | 1 | 23 | 0.273453 | SEC62 |
| Apoptosis | cpic04210 | 4 | 197 | 0.275472 | BCL2L1\|ITPR3\|RELA\|ERN1 |
| PPAR signaling pathway | cpic03320 | 2 | 77 | 0.280564 | ACSBG1\|ACADM |
| Other types of O-glycan biosynthesis | cpic00514 | 1 | 25 | 0.292549 | GXYLT2 |
| Inositol phosphate metabolism | cpic00562 | 2 | 80 | 0.295243 | PIK3C3\|PLCD1 |
| Cell adhesion molecules (CAMs) | cpic04514 | 4 | 204 | 0.296078 | CD4\|CD226\|SDC4\|CADM3 |
| Autophagy - animal | cpic04140 | 3 | 141 | 0.296841 | BCL2L1\|PIK3C3\|ERN1 |
| Other glycan degradation | cpic00511 | 1 | 26 | 0.301909 | ENGASE |
| Butanoate metabolism | cpic00650 | 1 | 27 | 0.311146 | ACAT2 |
| Glycosaminoglycan biosynthesis - heparan sulfate / heparin | cpic00534 | 1 | 27 | 0.311146 | EXT2 |
| Pentose phosphate pathway | cpic00030 | 1 | 28 | 0.32026 | RBKS |
| Cardiac muscle contraction | cpic04260 | 2 | 87 | 0.329293 | CACNB2\|CACNB4 |
| Purine metabolism | cpic00230 | 3 | 152 | 0.336552 | AMPD2\|PDE4B\|ADCY6 |
| Citrate cycle (TCA cycle) | cpic00020 | 1 | 30 | 0.338131 | SUCLG1 |
| Autophagy - other | cpic04136 | 1 | 30 | 0.338131 | PIK3C3 |
| ErbB signaling pathway | cpic04012 | 2 | 91 | 0.348554 | TGFA\|ABL1 |
| Mucin type O-glycan biosynthesis | cpic00512 | 1 | 32 | 0.355532 | GALNT8 |
| Focal adhesion | cpic04510 | 4 | 225 | 0.358739 | LAMC3\|COL6A3\|LAMC1\|PDGFD |
| Base excision repair | cpic03410 | 1 | 35 | 0.380784 | MPG |
| SNARE interactions in vesicular transport | cpic04130 | 1 | 36 | 0.38898 | STX1A |
| alpha-Linolenic acid metabolism | cpic00592 | 1 | 37 | 0.397067 | PLA2G3 |
| beta-Alanine metabolism | cpic00410 | 1 | 37 | 0.397067 | CNDP2 |
| Protein processing in endoplasmic reticulum | cpic04141 | 3 | 171 | 0.404653 | ERN1\|SEC62\|UBE2D1 |
| Pyruvate metabolism | cpic00620 | 1 | 38 | 0.405049 | ACAT2 |
| Linoleic acid metabolism | cpic00591 | 1 | 39 | 0.412924 | PLA2G3 |
| Nicotinate and nicotinamide metabolism | cpic00760 | 1 | 42 | 0.435934 | NAPRT |
| Glycine, serine and threonine metabolism | cpic00260 | 1 | 43 | 0.443402 | DMGDH |
| ABC transporters | cpic02010 | 1 | 47 | 0.472302 | ABCG4 |
| Carbon metabolism | cpic01200 | 2 | 119 | 0.476562 | ACAT2\|SUCLG1 |
| Tryptophan metabolism | cpic00380 | 1 | 50 | 0.492991 | ACAT2 |
| Steroid hormone biosynthesis | cpic00140 | 1 | 51 | 0.499706 | HSD11B2 |
| Oocyte meiosis | cpic04114 | 2 | 125 | 0.502001 | ITPR3\|ADCY6 |
| Cell cycle | cpic04110 | 2 | 129 | 0.518514 | ABL1\|E2F5 |
| Ferroptosis | cpic04216 | 1 | 56 | 0.531975 | STEAP3 |
| Cytosolic DNA-sensing pathway | cpic04623 | 1 | 58 | 0.544293 | RELA |
| FoxO signaling pathway | cpic04068 | 2 | 140 | 0.562009 | PLK3\|INSR |
| Pyrimidine metabolism | cpic00240 | 1 | 65 | 0.584919 | UCK2 |
| Drug metabolism - other enzymes | cpic00983 | 1 | 68 | 0.601205 | UCK2 |
| Regulation of actin cytoskeleton | cpic04810 | 3 | 240 | 0.625453 | PDGFD\|FGF9\|NCKAP1 |
| Ribosome biogenesis in eukaryotes | cpic03008 | 1 | 73 | 0.626945 | MDN1 |
| Insulin signaling pathway | cpic04910 | 2 | 158 | 0.626963 | INSR\|FASN |
| Wnt signaling pathway | cpic04310 | 2 | 168 | 0.659703 | NFATC1\|LGR6 |
| Salmonella infection | cpic05132 | 1 | 88 | 0.694645 | RELA |
| Adherens junction | cpic04520 | 1 | 93 | 0.71437 | INSR |
| Toll-like receptor signaling pathway | cpic04620 | 1 | 99 | 0.736371 | RELA |
| Progesterone-mediated oocyte maturation | cpic04914 | 1 | 99 | 0.736371 | ADCY6 |
| Tight junction | cpic04530 | 2 | 196 | 0.739179 | SCRIB\|RAP2C |
| TGF-beta signaling pathway | cpic04350 | 1 | 102 | 0.74673 | E2F5 |
| Melanogenesis | cpic04916 | 1 | 112 | 0.778415 | ADCY6 |
| Herpes simplex virus 1 infection | cpic05168 | 4 | 427 | 0.823948 | BCL2L1\|PTPN11\|RELA\|TYK2 |
| Spliceosome | cpic03040 | 1 | 132 | 0.830419 | U2AF2 |
| Lysosome | cpic04142 | 1 | 158 | 0.880267 | AP1S3 |
| mTOR signaling pathway | cpic04150 | 1 | 162 | 0.886513 | INSR |
| Necroptosis | cpic04217 | 1 | 181 | 0.912026 | TYK2 |
| Phagosome | cpic04145 | 1 | 222 | 0.949254 | PIK3C3 |
